# Intense Beauty Requires Intense Pleasure

**DOI:** 10.3389/fpsyg.2019.02420

**Published:** 2019-11-05

**Authors:** Aenne A. Brielmann, Denis G. Pelli

**Affiliations:** ^1^Department of Psychology, New York University, New York, NY, United States; ^2^Center for Neural Science, New York University, New York, NY, United States

**Keywords:** beauty, aesthetics, pleasure, anhedonia, depression

## Abstract

At the beginning of psychology, Fechner (1876) claimed that beauty is immediate pleasure, and that an object’s pleasure determines its value. In our earlier work, we found that intense pleasure always results in intense beauty. Here, we focus on the inverse: Is intense pleasure *necessary* for intense beauty? If so, the inability to experience pleasure (anhedonia) should prevent the experience of intense beauty. We asked 757 online participants to rate how intensely they felt beauty from each image. We used 900 OASIS images along with their available valence (pleasure vs. displeasure) and arousal ratings. We then obtained self-reports of anhedonia (TEPS), mood, and depression (PHQ-9). Across images, beauty ratings were closely related to pleasure ratings (*r* = 0.75), yet unrelated to arousal ratings. Only images with an average pleasure rating above 4 (of a possible 7) often achieved (>10%) beauty averages exceeding the overall median beauty. For normally beautiful images (average rating > 4.5), the beauty ratings were correlated with anhedonia (*r* ∼−0.3) and mood (*r* ∼ 0.3), yet unrelated to depression. Comparing each participant’s average beauty rating to the overall median (5.0), none of the most anhedonic participants exceeded the median, whereas 50% of the remaining participants did. Thus, both general and anhedonic results support the claim that intense beauty requires intense pleasure. In addition, follow-up repeated measures showed that shared taste contributed 19% to beauty-rating variance, only one third as much as personal taste (58%). Addressing age-old questions, these results indicate that beauty is a kind of pleasure, and that beauty is more personal than universal, i.e., 1.7 times more correlated with individual than with shared taste.

## Introduction

Beauty has fascinated humankind since ancient times, before Homer (see e.g., Hofstader and Kuhns, 1976), and was one of the first phenomena investigated in experimental psychology (e.g., Fechner, 1876; Lipps, 1906). In common understanding, the concept of beauty is central to aesthetics (Jacobsen et al., 2004; Brielmann and Pelli, 2018; Menninghaus et al., 2019a). Yet, beauty remains a controversial concept as the neuroaesthetics literature has yet to agree on a clear consistent definition.

The one aspect of beauty that philosophers, psychologists and neuroscientists agree on is that it fundamentally involves pleasure. Psychological theories increasingly acknowledge that pleasure plays a pivotal role in aesthetic experience and art appreciation (for reviews see: Jacobsen et al., 2004; Leder, 2013; Pelowski et al., 2016). Fechner (1876) claimed that “[t]he potential to immediately elicit liking and therewith pleasure always stays central for the term beauty also in its narrowest conception…” The fluency theory of aesthetic processing explicitly equates beauty with “aesthetic pleasure” (Reber et al., 2004). These approaches follow the philosophers in taking beauty to be a kind of pleasure (e.g., Plato, 390 BCE; Kant, 1790/2000; Hume, 1878; Santayana, 1896). For example, Plato tentatively defined beauty as pleasure through eye or ear, and Hume (1878) said that “pleasure and pain … are not only necessary attendants of beauty and deformity, but constitute their very essence.” Thus, many philosophers, psychologists, and neuroscientists have suggested that beauty is a kind of pleasure.

While we still lack proof for the necessity of pleasure for experiencing beauty, it is well-known that aesthetic liking and other sensory valuations engage the reward system, i.e., are bound to the experience of pleasure. Functional magnetic resonance (fMRI) studies shows that the appraisal of an object’s valence, i.e., the pleasure or displeasure it elicits, is key to aesthetic appraisal (for a meta-analysis see Brown et al., 2011). Other studies have shown that activity in the mesocorticolimbic reward circuit is key to the valuation of diverse perceptual pleasures such as looking at attractive faces (Chelnokova et al., 2014) or erotic pictures (Buchel et al., 2018), tasting something sweet (Eikemo et al., 2016), as well as listening to music (Mallik et al., 2017). This work strongly suggests that pleasure, i.e., activity in the reward circuit, is necessary for beauty.

But what separates beauty from other pleasures? Valentine observed that “toffee may give keen pleasure to the sense of taste, but we could hardly call it ‘beautiful”’ (Valentine, 1962, pp. 3f). While some research suggests that the key difference between beauty and other pleasures lies in the involvement of cognitive factors (e.g., Brielmann and Pelli, 2017; Skov, 2019a, b), a bolder viewpoint is that Valentine was wrong. People do call candy beautiful if they experience above-threshold pleasure from it (Brielmann and Pelli, 2017), provoking the hypothesis that any above-threshold pleasure qualifies as a beauty experience (Brielmann and Pelli, 2018).

Apart from pleasure, arousal has been mentioned as crucial for the experience of beauty. Berlyne in his seminal book *Aesthetics and Psychobiology* (1973) suggested that pleasure and aesthetic value have the same inverted U-shaped relationship with arousal: Highest aesthetic value, or beauty, should arise at intermediate levels of arousal. Armstrong and Detweiler-Bedell’s (2008) theory posits that beauty is characterized by intense pleasure and increased arousal, similar to a model developed by Pelowski and Akiba (2011).

### Looking at Anhedonia, Mood, and Depression to Test the Link Between Beauty and Pleasure

This short overview showed that the notion of pleasure is a common denominator of most accounts of beauty, while some also include arousal. Both components – pleasure and arousal – have been identified as usually independent components of affect (Posner et al., 2005). Within this framework, pleasure is conceptualized as *valence*, where the positive side of the scale is pleasure and the negative side displeasure. Valence and arousal are routinely used to characterize the emotional experience of stimulus sets (Lang et al., 1997; Bradley and Lang, 1999; Brielmann and Stolarova, 2015; Kurdi et al., 2017). Thus, as a first step toward a study of the link between emotion and beauty, we here investigate how judgments of beauty relate to judgments of valence and arousal.

Our brief review above shows wide support for the philosophical claim that the feeling of beauty is a kind of pleasure. This assumption is justified by studies that find a strong positive relation between beauty and pleasure ratings for diverse stimuli (Vartanian et al., 2013; Brielmann and Pelli, 2017; Brielmann et al., 2017) and by imaging studies that demonstrate a strong link between activity in reward pathways and aesthetic liking (e.g., Chelnokova et al., 2014; Sachs et al., 2016; Skov, 2019a). If pleasure is indeed the main manifestation of beauty, then people unable to experience pleasure should also be unable to experience beauty. Clinically, the inability (or greatly decreased ability) to experience pleasure is called *anhedonia*.

Anhedonia is one of the main symptoms of major depressive disorders according to both the *Diagnostic and Statistical Manual of Mental Disorders* (DSM; American Psychiatric Association, 2013) and *International Classification of Diseases* (ICD; American Medical Association, 2015). And there are autobiographical reports that beauty vanishes during depression. For example, Lewis (1961) recounts his recovery from grief over the death of his wife, saying “Today I have been revisiting old haunts, taking one of the long rambles that made me so happy in my bachelor days. And this time the face of nature was not emptied of its beauty and the world didn’t look (as I complained some days ago) like a mean street.”

Does depression prevent feeling beauty? It’s important to distinguish the clinical diagnosis of depression (as assessed by questionnaires like the PHQ-9) from the degree of “depression” as a component of daily mood, and to note the neuronal dissociation between anhedonia and depression. Thus, the DSM link between depression and anhedonia might not extend to feeling “depressed” as used in casual language.

Three lines of evidence suggest that anhedonia is related to the ability to experience beauty. One, several studies document the existence of a type of anhedonia that only affects aesthetic pleasure derived from music (Mas-Herrero et al., 2014, 2018; Martínez-Molina et al., 2016, 2019; Mallik et al., 2017). Two, studies that manipulated opiod receptor activity and therewith the pleasure response in the reward circuits demonstrated that such an induced anhedonic state diminishes the pleasure experienced from faces (Chelnokova et al., 2014), sweet taste (Eikemo et al., 2016), erotic pictures (Buchel et al., 2018), and music (Mallik et al., 2017). Three, anhedonia – but not depression – has been linked to decreased activation in the ventromedial prefrontal cortex (vmPFC) in response to happy memories (Keedwell et al., 2005). This same brain region is consistently more highly activated compared to baseline when experiencing beauty from various stimuli (Kawabata and Zeki, 2004; Ishizu and Zeki, 2011; Zeki et al., 2014).

These findings reinforce the hypothesis that anhedonia decreases the ability to experience beauty. Testing this hypothesis provides another test of the presumed link between beauty and pleasure.

### Current Study

In sum, psychological and philosophical theories suggest that the experience of beauty is associated with pleasure. To test these theories, we used an open-access database containing 900 images (OASIS^1^; Kurdi et al., 2017). The database includes average pleasure and arousal ratings of the images by a United States online population. In our present study, we add ratings of beauty intensity from a very similar population (*N* = 757). We make these beauty ratings openly accessible to facilitate and encourage more study of beauty^2^.

The current study thus had four aims: (1) to provide a first set of ratings (*N* = 757) of beauty for a large image set, the 900 OASIS images, (2) to investigate how much of the variance of these beauty ratings can be attributed to shared vs. idiosyncratic taste, (3) to describe the relation between beauty and two components of affect: valence and arousal, and (4) to further test the link between beauty and pleasure by describing the relation between beauty and several indicators of low pleasure: anhedonia, low mood, and depression.

## Materials and Methods

### Participants

We obtained data from 757 participants (367 men, 387 women, 3 “other,” *M*_age_ = 38.4 years, *SD* = 12.7, range = 18–84) from Amazon mechanical Turk (mTurk) to “Rate images on how beautiful they are” in exchange for $1. All participants consented to participate according to a consent form approved by the NYU UCAIHS (university committee on activities involving human subjects; IRB-FY2016-404) by checking a box in the online form. We collected our data in mid-July 2017.

**FIGURE 1:**
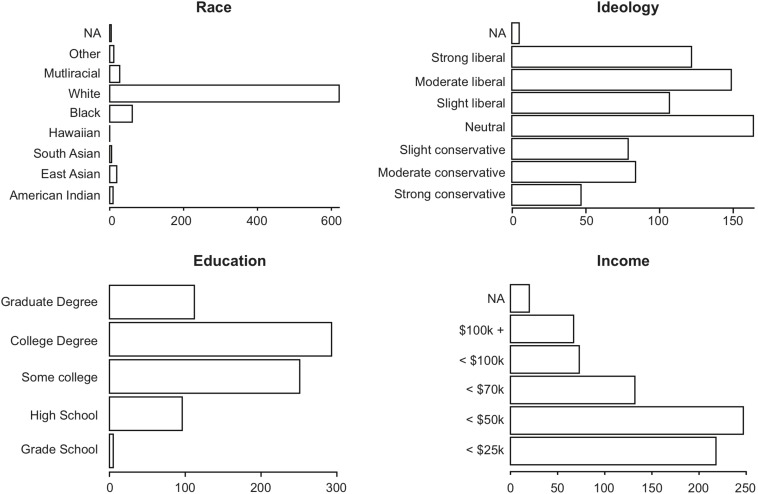
Distributions of self-reported race, ideology, highest educational attainment, and annual household income.

The sample size was chosen to match that of the Kurdi et al. (2017) study to maximize comparability between studies. We used exactly the same recruitment method and inclusion criteria as Kurdi et al. (2017) did. mTurk workers with an approval rate of at least 90%, who have completed at least 50 HITs, and reside in the United States. Study completion took on average 30.9 ± 10.9 min (*M* ± *SD*). We also assessed the same demographic variables as Kurdi et al. (2017) did and find that our sample exhibits the same distribution regarding all of them (age, gender, income, political orientation, general education, ethnicity). Demographics are displayed in Figure 1.

### Stimuli and Procedures

We used all 900 OASIS images in their original size (500 × 400 px) as stimuli (Kurdi et al., 2017). The images are in four exclusive categories: 134 “animals,” 200 “objects,” 346 “people,” and 220 “scenes.” It is worth noting that the “people” category of the 900 OASIS images has 346 images, including 41 faces, and 305 scenes of one or more people in various situations, including 22 romantic couples and 45 images of individual men and women labeled as “nude,” i.e., a total of 67 images with potential erotic connotation. These images are dramatic, unlike the expressionless portraits typically used in studies of human beauty or attractiveness.

We randomly divided the 900 images into four subsets (1, 2, 3, 4), each having 225 images, to reduce the number of images each participant would rate. Each participant saw and rated one of the four 225-picture subsets. 368 participants rated images from subset 1, 140 from 2, 145 from 3, and 104 from 4.

The survey was set up using the online service Qualtrics. Instructions were adapted from those for arousal ratings of the OASIS images (Kurdi et al., 2017) and are provided in full in Supplementary File S1. One of the goals of this study is to discover the relation between beauty and pleasure, so observers are asked to rate both. The instructions do not define “beauty,” because that would defeat our purpose for the reasons that we spelled out in an earlier paper (Brielmann and Pelli, 2017). All reasonable definitions of beauty that we know (e.g., Kant or Santayana) specify a strong connection between beauty and pleasure. Providing such a beauty definition to our observers would have revealed that hypothesized connection to them, which might bias them to produce correlated ratings of pleasure and beauty. Since such a bias is hard to assess and discount, we take care not to introduce it in the first place. Thus, the observer’s instructions do not define “beauty.”

After informed consent, the first screen advised participants that there are no right or wrong answers. The second screen explained how to use the beauty scale, including its middle and end points. The third screen emphasized that ratings should reflect only the participant’s feelings, regardless of the goodness or badness of the image content.

Participants then proceeded to rate the intensity of the feeling of beauty elicited by each picture. Each trial presented one image, at the top center of the screen, with the rating scale below. Participants advanced at their own pace by clicking a separate “>>” button in the right corner of the screen. After rating all 225 images, participants were asked about their mood, whether they had seen any file name of the images (file names were automatically displayed as the “alternate caption” due to slow internet connections when images took longer than a few seconds to load), and whether these influenced their ratings. Next, participants filled out the temporal-experience-of-pleasure scale (TEPS; Gard et al., 2006) and, on a separate screen, the PHQ-9 (Martin et al., 2006). (The scales are explained below). Finally, participants were asked basic demographic questions: age, gender, income, race, ZIP codes of current and longest residency, and political orientation. All of these assessments (mood, anhedonia, and depression) were made only after finishing the image ratings, so the observers rated pleasure and beauty with no awareness of our interest in mood, anhedonia, and depression.

The first question after rating beauty is mood. We assessed current mood with a single question asking how the participant currently felt on a scale from 1 (miserable) to 100 (excellent). This was included as a simple measure of current emotional state, complementing our TEPS and PHQ-9 measures of trait-like characteristics.

The TEPS scale provides scores for the long-term ability to experience pleasure and thus served as an assay for anhedonia (Rizvi et al., 2016). It has been widely endorsed as a tool to assess anhedonia in clinical and non-clinical populations (Horan et al., 2006; Chentsova-Dutton and Hanley, 2010; Treadway and Zald, 2011; Der-Avakian and Markou, 2012; Liu et al., 2012; Rømer Thomsen et al., 2015; Rizvi et al., 2016). We chose TEPS rather than another measure of anhedonia due to its brevity. The TEPS includes two subscales. The *anticipatory pleasure* scale measures the pleasure experienced in anticipation of a positive event while the *consummatory pleasure* scale measures the in-the-moment pleasure in response to an event. Each item is a statement about an experience and asks the participant to rate how true each one is for her or him. Anticipatory pleasure is measured with items like “I get so excited the night before a major holiday I can hardly sleep,” consummatory pleasure with items like “The smell of freshly cut grass is enjoyable to me.”

The PHQ-9 is a questionnaire developed to assess severity of depression in clinical and non-clinical populations. Its nine items exclusively assess the diagnostic symptoms for major depression listed in the DSM-IV-TR. For each item, participants indicate how often in the past 2 weeks they experienced states like “Little interest or pleasure in doing things” on a scale from 0 (never) to 3 (nearly every day). Thus, the PHQ-9 provides a graded measure of the severity of depression as well as specific cut-off values for depression diagnoses (Kroenke et al., 2001; Kroenke and Spitzer, 2002; Martin et al., 2006).

## Results

### Distribution of Beauty Ratings

The 900 images were split into four sets of 225 images, and each image received one rating from each participant assigned to its set. Thus, 368 ratings were obtained for images of the first picture set and 140, 145, and 104 ratings per image for subsets 2, 3, and 4, respectively.

**FIGURE 2:**
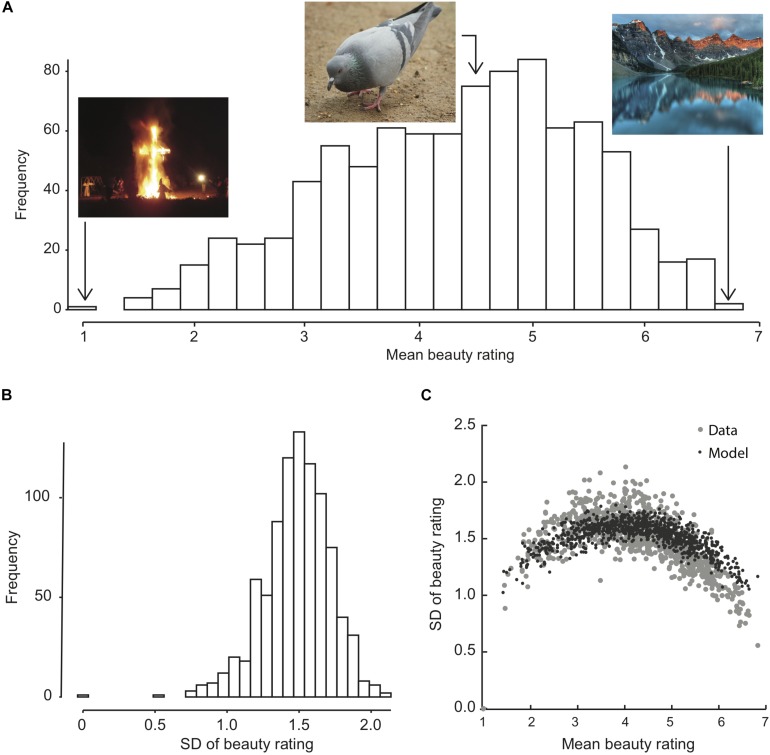
Distribution, across images, of the mean and *SD* across observers of the beauty rating for each image **(A,B)**, and their relation **(C)**. Example images in **(A)** show the lowest and highest rated images as well as one of the images receiving the median average rating of 4.45. OASIS allows for the free use of images in online and offline research studies as they are not subject to copyright restrictions. **(C)** Compares the data (gray dots) with a model prediction (black dots) is based on 190 simulated beauty ratings per image (as many as the average number of participant ratings per image), drawn from a normal distribution with a mean equal to the mean beauty of each image and an *SD* of 1.7, and rounded to the nearest allowed integer response 1–7.

For each image, we calculated the mean and *SD* of its beauty rating across observers. The distribution of these statistics, along with examples of median, highest, and lowest rated images are shown in Figure 2. Participants used the entire range of the integer scale: 1–7. Mean beauty ratings per image ranged from 1.0 to 6.83. The distribution of means was not normal according to Shapiro–Wilk test for normality but skewed to the left, *W* = 0.98, *p* < 0.001, parameter estimates for skewed normal distribution, ω = 1.68, α = −2.39 (Azzalini, 2018).

There is a quadratic relationship between *SD* and mean of the beauty rating (Figure 2C), *R*^2^_adj_ = 0.61, *p* < 0.001. This was expected, because the beauty scale (like the arousal scale from which the instructions were adapted) is bounded at both ends, which tends to reduce the variance near the ends. To quantify this effect, we ran simulations with a model that simulates a beauty report as mean beauty plus normally distributed noise with *SD* = 1.7, rounded to the nearest allowed integer rating 1–7. This model roughly replicates the observed distribution of *SD*s. The main deviation from the observed data was that the observed *SD*s for the most beautiful images were lower than predicted by the model. The model assumes that the internal analog responses for all images (before rounding) have the same *SD*.

### Beauty vs. Pleasure and Arousal

**FIGURE 3:**
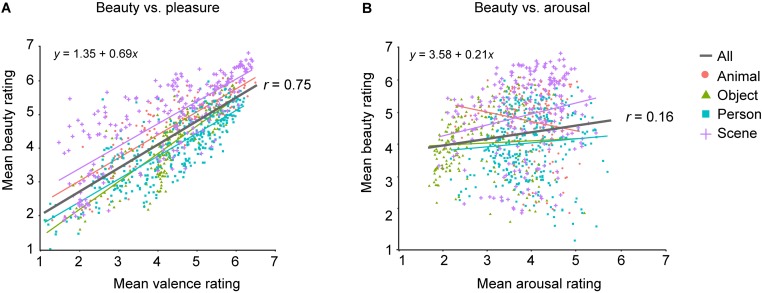
Linear relations between mean beauty and pleasure **(A)** and arousal ratings **(B)**. Each point represents the mean rating for one image across all participants. Each point’s color and shape indicate the image category: red circles for animals; green triangles for objects; blue squares for persons; violet crosses for scenes. Lines represent linear fits. Black lines are fit across all image categories. Note that quadratic fits with arousal as predictor explained hardly any more variance in beauty ratings than the linear fits do. For arousal, all linear *R*^2^_adj_ = 0.00, whereas the maximum quadratic *R*^2^_adj_ = 0.04. Mean pleasure and arousal ratings were obtained from http://www.benedekkurdi.com/oasis#oasis.

Figure 3 displays the relationship between the mean beauty ratings in our study and the mean valence and arousal ratings per image obtained by Kurdi et al. (2017). While ratings of pleasure-displeasure are labeled as “valence” in the OASIS database, we will refer to that as “pleasure,” to minimize jargon. Beauty was highly positively correlated with pleasure, *r*(898) = 0.75, *p* < 0.001, 95% CI [0.73, 0.78]. When using general models to explain mean beauty by pleasure, there was little difference between the linear and quadratic models, difference in corrected Akaike Information Criterion AICc = 8.77, both *R*^2^_adj_ = 0.57. Unlike that with pleasure, the positive correlation between beauty and arousal ratings was very weak, *r*(898) = 0.16, *p* < 0.001, [0.09, 0.22]. Introducing a quadratic term for predictions of beauty from arousal ratings slightly improved the model fit, difference in AICc = 10.46, quadratic *R*^2^_adj_ = 0.04, vs. linear *R*^2^_adj_ = 0.02.

Estimates by the linear models are shown in Figure 3. Beauty was moderately well explained by pleasure in a linear way (*R*^2^_adj_ = 0.57). In contrast, arousal ratings accounted for very little of the variance in beauty (*R*^2^_adj_ = 0.04) and the relation between beauty and arousal was best described by an inverted U-shape: Beauty is depressed at the extremes, equivalent to a very weak version of Berlyne’s (1971) claim.

We repeated these analyses for images in each of the four pre-defined image categories. Whereas the positive correlation between beauty and pleasure was evident in all image categories, all *r* ≥ 0.78, all *p* < 0.001, a positive linear relation between beauty and arousal was evident only for scenes, *r*(218) = 0.21, *p* = 0.001, 95% CI [0.08, 0.34], and not for objects, persons (both *p* ≥ 0.122) or animals. For animals, the insignificant correlation is reversed, *r*(132) = −0.16, *p* = 0.060, [−0.32, 0.01]. There was no difference in the goodness of fit for linear vs. quadratic models in predicting beauty from arousal for animal pictures, both *R*^2^_adj_ = 0.02, difference in AICc = 0.93, or scenes, both *R*^2^_adj_ = 0.04, difference in AICc = 1.96. Beauty was slightly better predicted from arousal by a quadratic model for persons, linear *R*^2^_adj_ = 0.00 vs. quadratic *R*^2^_adj_ = 0.04, difference in AICc = 11.08, and objects, linear *R*^2^_adj_ = 0.00 vs. quadratic *R*^2^_adj_ = 0.18, difference in AICc = 39.88. Note, however, that both quadratic models still explain very little variance.

This pattern of results affirms a robust positive correlation between beauty and pleasure. Yet, despite the high positive correlation between beauty and pleasure (*r* = 0.75), it is unlikely that observers conflate beauty and pleasure. For instance, the image “Tornado4” received an average beauty rating of 5.66 ± 1.34 and an average pleasure rating of merely 2.70 ± 1.36, whereas the image “Fireworks4” received almost the same beauty (5.67 ± 1.29), but a much higher pleasure rating (5.95 ± 0.91), more than double. Figure 3A illustrates that unpleasant images can be beautiful, but, at least in this image set, very pleasant images never fail to be beautiful, extending and confirming our original report (Brielmann and Pelli, 2017).

We find no general relation between arousal and beauty. If anything, beauty is weakly related to arousal in an inverted U-shaped manner [a very weak version of what Berlyne (1971) claimed], but this held only for images of persons and objects, while there was a positive linear relation between arousal and beauty for scenes (*r* = 0.21).

All results reported below are based on ratings of all 225 images made by each participant. To rule out that fatigue due to the number of trials influenced our results, we repeated the main analyses with the first 100 ratings each participant gave. The results are equivalent to the ones reported below and can be found in Supplementary File S3.

### Gender Effects

**FIGURE 4:**
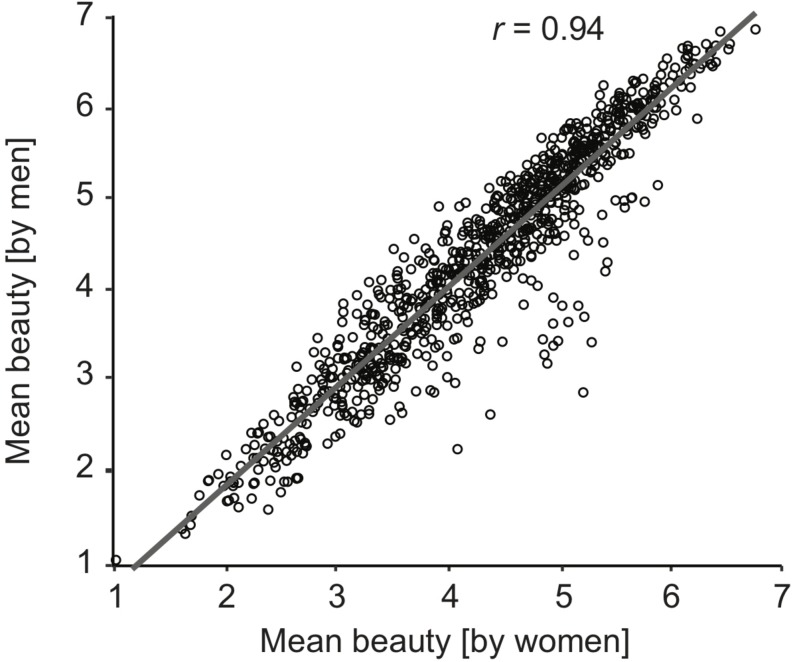
Mean beauty rating by men (vertical axis) vs. women (horizontal axis) for each image are very highly correlated. One coherent subset of images (mostly “nude couples”) were rated higher in beauty by women than by men. Out of 14 images of “nude couples” in the total image database, 12 were rated more than one point higher in beauty by women than by men.

Overall, men’s and women’s average beauty ratings per image were highly correlated, *r*(898) = 0.94, *p* < 0.001, 95% CI [0.93, 0.95]. However, the scatterplot illustrating this finding (Figure 4) shows that there was a subset of images that was rated higher in beauty by women than by men, whereas no group of images stands out as more highly rated by men. Whereas 16 images were rated more than one point higher by women than by men, only one image (“Horseracing1”) was rated one point higher by men than women. The images that were rated considerably (more than one point) higher by women mostly depicted people (16/20 = 80%), while only 38% of OASIS images are images of people. Notably, most of these person images showed couples. Out of 14 images of “nude couples” in the total image database, 12 were rated more than one point higher in beauty by women than by men. The differences in beauty ratings between men and women were unrelated to the images’ arousal and pleasure, both *p* ≥ 0.1. We report all further, minor demographic effects in Supplementary File S2.

### Beauty vs. Anhedonia, Mood, and Depression

The participants’ moods were assessed by asking them how they currently felt on a scale from 1 (miserable) to 100 (excellent). We measured degree of depression with the PHQ-9 questionnaire and anhedonia with the temporal experience of pleasure scale (TEPS). The TEPS consists of two scales, one assessing anticipatory and one consummatory pleasure. Higher scores on the PHQ-9 indicate greater depression; lower scores on the TEPS scales indicate greater signs of anhedonia.

**FIGURE 5:**
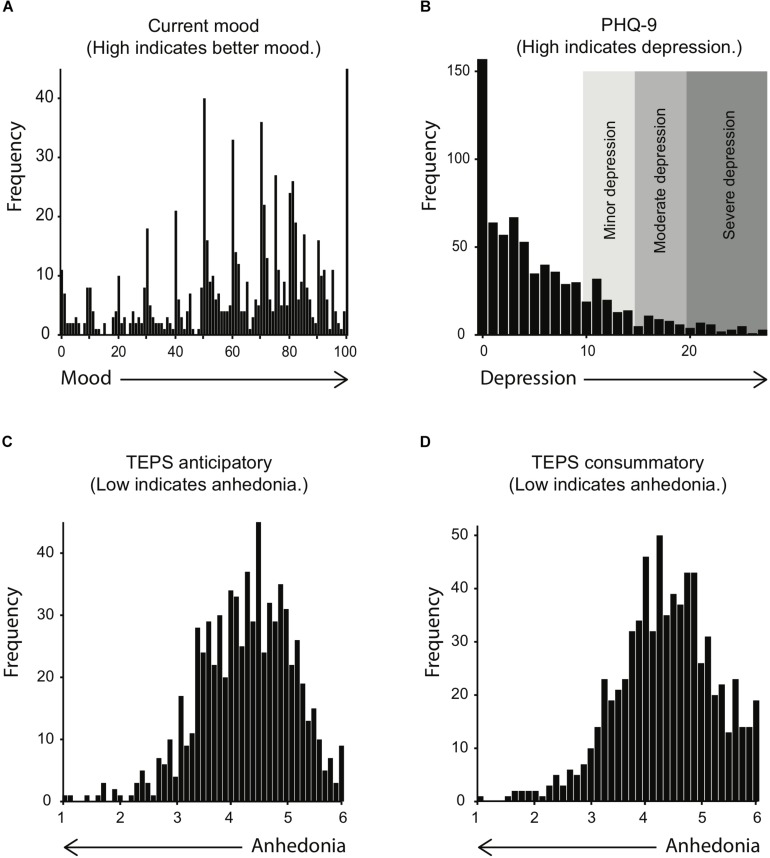
Histograms of mood **(A)**, PHQ-9 **(B)**, TEPS anticipatory **(C)**, and TEPS consummatory scores **(D)**. Shaded areas in **(B)** indicate provisional diagnoses based on the PHQ-9 scores, as specified by the PHQ-9 manual (http://www.cqaimh.org/pdf/tool_phq9.pdf).

Internal consistency was excellent for the PHQ-9, α = 0.91, and sufficient for both anticipatory, α = 0.74, and consummatory TEPS scales, α = 0.68. (Mood is a one-question score, so no internal consistency can be calculated for it). The distribution of mood, TEPS, and PHQ-9 scores is shown in Figure 5. All measures have skewed distributions. As expected for a non-clinical sample, participants were most likely to have high anticipatory and consummatory TEPS scores, and low PHQ-9 scores, indicating healthy experience of pleasure and absence of depression. Accordingly, mood scores were rather high, with peaks at round numbers on the scale from 1 to 100. Yet, the distributions also show that our sample was large enough to include a considerable number of participants with PHQ-9 scores that indicate mild (*N* = 39) to severe depression (*N* = 31), as well as TEPS scores that can be interpreted as a sign of anhedonia (*N* = 40 with TEPS consummatory scores < 3, and *N* = 47 with TEPS anticipatory scores < 3).

Temporal-experience-of-pleasure scale scores were moderately positively related to mood, *r*(724) = 0.31, *p* < 0.001, 95% CI [0.23, 0.37] for TEPS anticipatory, and *r*(724) = 0.25, *p* < 0.001, [0.18, 0.32] for TEPS consummatory. In a complementary way, both TEPS scores were negatively associated with PHQ-9 scores, *r*(706) = −0.28, *p* < 0.001, [−0.35, −0.21] for TEPS anticipatory, and *r*(706) = −0.20, *p* < 0.001, [−0.27, −0.12] for TEPS consummatory. As expected, mood was also negatively correlated with PHQ-9 scores, *r*(706) = −0.26, *p* < 0.001, [−0.33, −0.19]. TEPS anticipatory and consummatory scores were positively correlated with one another, *r*(724) = 0.63, *p* < 0.001, [0.58, 0.67]. All the pairwise correlations appear in Table 1. The first beauty row shows the correlations between scores and mean beauty ratings across all images, and the second shows correlations across only the images with higher-than-median beauty ratings.

**TABLE 1:** Pearson correlations between beauty rating (observer means across images) and measures of anhedonia (TEPS anticipatory, TEPS consummatory), mood, and depression (PHQ-9).

			**Anhedonia**			**Depression**
					
***r***	**Beauty**	**Beauty^∗^**	**TEPS**	**TEPS**	**Mood**	**PHQ-9**
			**ant.**	**con.**		
Beauty	1	–	0.29	0.23	0.20	–0.02
Beauty^∗^		1	0.36	0.34	0.27	–0.05
TEPS anticipatory			1	0.63	0.31	–0.28
TEPS consummatory				1	0.25	–0.20
Mood					1	–0.26
PHQ-9						1

*^∗^The second row is restricted to only the images whose average beauty rating is higher than median. See main text for detailed statistics.*

#### A Linear Model

**FIGURE 6:**
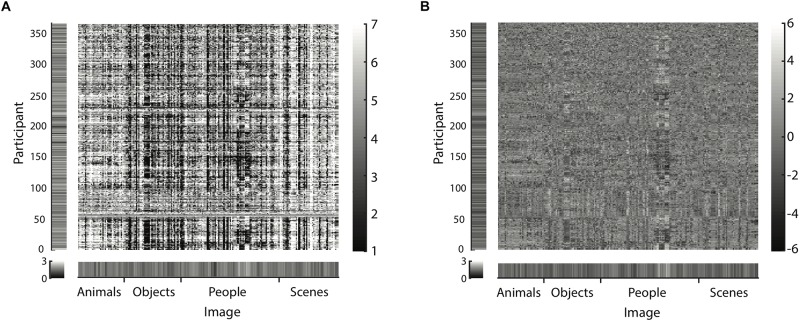
Exemplary matrix of raw data **(A)** and residuals for the model specified by Eq. 2 **(B)** for image set 1 (225 images). Matrices for all other image sets are available as Supplementary Figures S1, S2. Data and residuals are sorted according to image category and name on the horizontal axis. Along the vertical axis, participants were sorted according to correlations between residuals: The first observer displayed at the bottom was picked randomly; for the next displayed participant, we picked from the remaining participants the one most correlated with the current one; and so on. Lighter areas indicate higher ratings **(A)** or residuals **(B)**, darker areas lower ones. Margins indicate the *SD* of beauty ratings **(A)** and residuals **(B)** per participant along the vertical axis and per image along the horizontal axis. The salient vertical and horizontal stripes in **(A)** reveal similar ratings across images and across participants (raters). The apparently random distribution of residual values in **(B)** suggests that the model predictions have relatively little systematic error.

To assess how anhedonia, mood, and depression influence the beauty experience, we fit a linear model to predict individual participants’ beauty ratings for each image from the observer and image averages,

(1) $$B_{i,0}=-4.279+0.992\,{\bar{B}}_{i}+0.984\,{\bar{B}}_{o}$$

where *i* = image index, and *o* = observer index. Each image’s mean beauty ${\bar{B}}_{\text{i}}$ is a moderately good predictor of its beauty rating, all by itself explaining 36% of the variance. Observer bias (mean beauty rating per participant, ${\bar{B}}_{\text{o}}$) alone explains 14% of the variance. Together, they account for 49% of the variance. The detailed results of this regression are shown in Eq. 1 and Table 2. Figure 6A illustrates this result that averages per image and participant already explain a substantial amount of variance of beauty ratings. The raw data matrix, exemplarily shown for the first data set, is dominated by clear vertical (image-related) and horizontal (observer-related) stripes of homogenous ratings.

**TABLE 2:** Results for the linear regression specified in Eq. 1.

	**β**	***SE***	***p***
Intercept	–4.279	0.025	< 0.001
${\bar{B}}_{\text{i}}$	0.992	0.003	< 0.001
${\bar{B}}_{\text{o}}$	0.984	0.005	< 0.001

#### A Non-linear Model With Interactions

Thus, the linear model of Eq. 1 accounts for nearly half the variance. We were unable to account for much more variance by going to a non-linear model that allows for interactions.

We were particularly interested in how anhedonia, mood, and depression influence beauty ratings differentially depending on the overall average image beauty. The regression in Eq. 2 below predicts beauty ratings based on the interaction of mean image beauty and anhedonia, mood, and depression, accounting for individual response biases. It takes the interaction between mean image beauty and all predictors into account while omitting all further interactions between those since they are (a) correlated with each other, (b) we were specifically interested in the unique interactions of anhedonia, mood, and depression with image beauty, and (c) we had no predictions that would aid the interpretation of interactions as complex as three- or higher-way interactions of continuous variables.

(2) $$\begin{array}{ccc} B_{io} & = & -2.696+0.616{\bar{B}}_{i}+0.979{\bar{B}}_{0}-0.005mood_{o}\\ & & -0.180TEPS_{ant,o}-0.109TEPS_{con,o}-0.003PHQ_{o}\\ & & +{\bar{B}}_{i}\times (0.001mood_{o}+0.037TEPS_{ant,o}\\ & & +0.033TEPS_{con,o}+0.001PHQ_{o}) \end{array}$$

**FIGURE 7:**
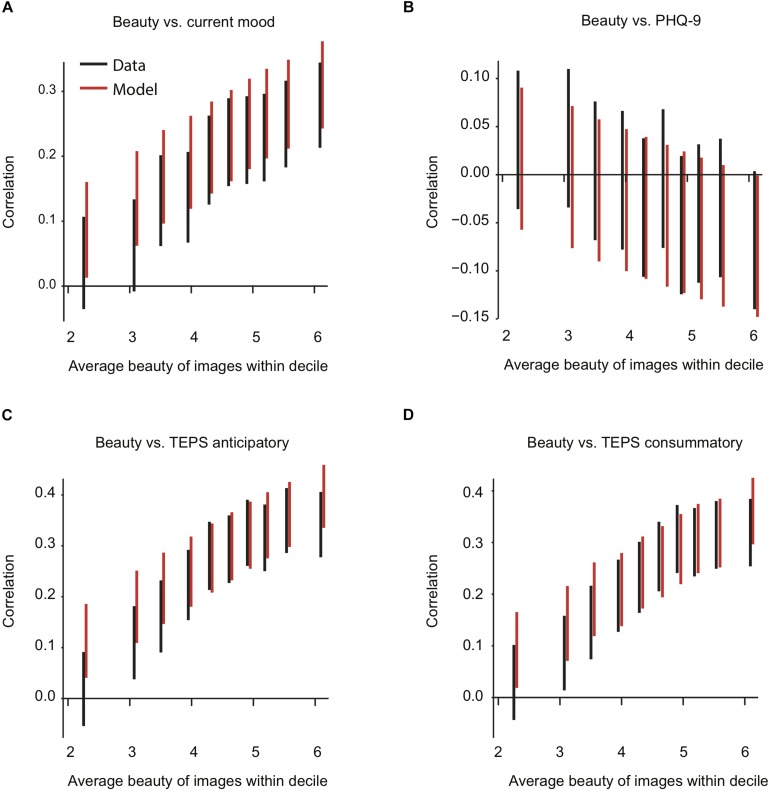
Vertical 95% confidence interval (CI) of the correlation coefficient of average beauty rating vs. mood **(A)**, PHQ-9 **(B)**, TEPS anticipatory **(C)**, and TEPS consummatory score **(D)**. Each line represents the CI for one decile. Horizontally, each CI is placed at the average beauty rating within its decile. Black lines represent the data, red lines model predictions based on Eq. 2. The first decile is the 10% of images that were rated lowest in beauty intensity, and so on. Model predictions are shifted rightward to avoid occlusion.

This model explains hardly any more variance than does Eq. 1, 49.08% vs. 48.90%, *p* < 0.001. However, it’s a useful result because it captures the effects of mood and anhedonia (and noneffect of depression), as we will show below (Figure 7). The regression coefficients for Eq. 2 are shown in Table 3. Unsurprisingly, beauty ratings increase with increasing image beauty. The model reveals important interactions: the increase of beauty ratings with mean beauty is amplified with heightened mood and TEPS scores (i.e., less anhedonic). That is, the higher a participant’s mood or less anhedonic, the more their beauty ratings increased with increasing mean beauty. Conversely, for participants with higher anhedonia or lower mood, beauty ratings differ less between on average low or intensely beautiful images. Beauty ratings are overall unrelated to depression (*p* = 0.191).

**TABLE 3:** Results for the linear regression specified in Eq. 2.

	**β**	***SE***	***p***
Intercept	–2.696	0.088	< 0.001
${\bar{B}}_{\text{i}}$	0.616	0.019	< 0.001
${\bar{B}}_{\text{o}}$	0.979	0.005	< 0.001
Anhedonia *TEPS*_ant,o_	–0.180	0.021	< 0.001
Anhedonia *TEPS*_con,o_	–0.109	0.020	< 0.001
*Mood*_o_	–0.005	0.001	< 0.001
Depression *PHQ*_o_	–0.003	0.002	0.191
${\bar{B}}_{\text{i}}$ × *mood_o_*	0.001	< 0.001	< 0.001
${\bar{B}}_{\text{i}}$ × *TEPS*_ant,o_	0.037	0.005	< 0.001
${\bar{B}}_{\text{i}}$ × *TEPS*_con,o_	0.033	0.004	< 0.001
${\bar{B}}_{\text{i}}$ × *PHQ_o_*	0.001	0.001	0.142

*Note that all the terms are significant except those for depression: the PHQ-9 score and its interaction with mean image beauty. Residuals for this model are shown in Figure 6B and Supplementary Figure S2.*

#### Unpacking Interactions Within Image Bins of Varying Beauty

Looking further for non-linear interaction of mean beauty vs. anhedonia, mood, and depression, we binned images into deciles according to their average beauty rating across participants ${\bar{B}}_{\text{i}}$. Within each bin, we calculated each participant’s average beauty rating. Then, we correlated these averages with participants’ scores on the TEPS, PHQ-9, and mood scale. We computed one correlation for each beauty decile. The 95% confidence intervals for all obtained correlation coefficients are shown in Figure 7. Overall, the results were equivalent to the regression (see section “A non-linear model with interactions”), as illustrated by the red lines representing the outcomes of the same analysis done on the predictions of the model specified in Eq. 2. This shows that, albeit small, the interaction between average beauty and anhedonia does produce an observable, distinct pattern of rating changes. The same is true for beauty and mood. These patterns are visible in the data and predicted by the model presented above.

In line with the findings from the linear regression, depression was hardly associated with a change in beauty ratings. PHQ-9 scores tend to correlate negatively with beauty ratings in the top decile only, i.e., for the most beautiful images, *r*(737) = −0.07, *p* = 0.06, 95% CI [−0.14, 0.00], but even this CI still includes zero (Figure 7B).

In contrast, we again find that beauty is consistently associated with the TEPS scores and mood (Figures 7A,C,D). The higher the beauty ratings, the stronger the positive association with TEPS or mood. Thus, the more beautiful the images were for the average population, the stronger the association between the beauty rating and anhedonia and high mood. Anhedonia and mood did not affect ratings for the images in the lowest beauty decile. Taken together, the results indicate that anhedonia and low mood mostly impair the ability to experience intense beauty. They do not dampen low beauty ratings.

#### High Beauty Ratings Generally Occur Only When Pleasure Is Intense

**FIGURE 8:**
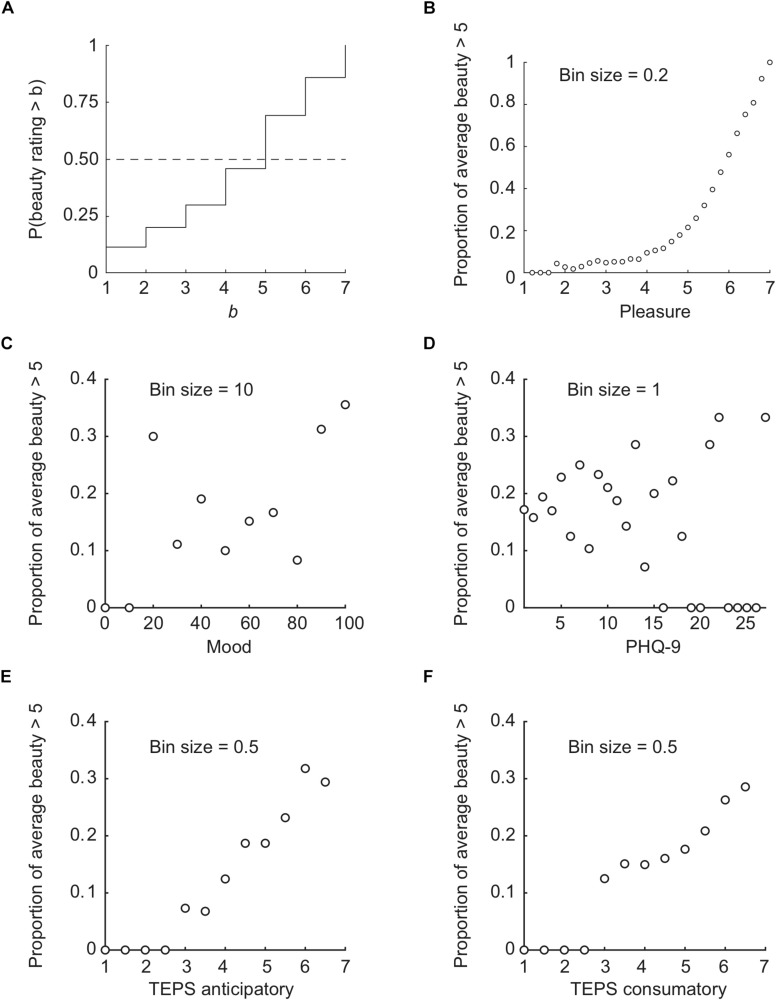
Cumulative probability distribution of beauty ratings **(A)**, and proportion of above-median average beauty per image **(B)** or participant **(C–F)** conditional on average pleasure **(B)**, mood **(C)**, PHQ-9 **(D)**, TEPS anticipatory **(E)**, and TEPS consummatory **(F)** scores. **(D)** Note that for the participants within the PHQ-9 bins with no above-median beauty ratings (0% points in **D**), the maximum TEPS anticipatory score did not exceed 3.9 and the maximum TEPS consummatory score did not exceed 4.4, i.e., they not only had high depression scores but also TEPS scores indicating anhedonia.

To substantiate our claim that intense beauty requires intense pleasure, we also looked at the proportion of above-median beauty ratings in relation to pleasure, mood, depression, and anhedonia. Figure 8A shows the cumulative probability distribution for beauty ratings. The intersection of the dashed line (50% probability) with the solid stair-like line indicates that the median beauty rating is 5. Here we refer to higher than median beauty ratings as “high.” Figures 8B–F show how the frequency of high beauty ratings depends on other measures.

First, the average pleasure and beauty ratings per image show that only images with an average pleasure rating of 4.5 also receive high (i.e., >5) average beauty ratings often (>10%; see Figure 8B). Second, the most-anhedonic participants (i.e., having TEPS scores below 3) never give high beauty ratings, while the least-anhedonic participants (i.e., with TEPS scores of 6 or more) give high beauty ratings to ∼30% of images (Figures 8E,F). Both findings substantiate the conclusion that intense beauty rarely occurs without high pleasure. In contrast, people with low mood (Figure 8C) or high depression (Figure 8D) do give high beauty ratings with a substantial frequency that seems to be independent of mood and depression (*r* not significantly different from 0).

These findings complement earlier results from our lab (Brielmann and Pelli, 2017) that showed that various stimuli (images, sucking candy, touching a teddy bear) are called “beautiful” whenever the pleasure of experiencing them exceeded a threshold pleasure of 4.3 on a 1–10 scale. Here, we find that only images with average pleasure of at least 4.5 on a 1–7 scale yield high average beauty ratings more than rarely.

### Beauty Variance Due to Differences Within and Across Observers

First, we replicated the reliability analyses of OASIS images by Kurdi et al. (2017). We calculated interrater reliabilities using a resampling method. Then we randomly generated 1,000 split halves of all beauty ratings, calculated the correlation between beauty ratings of the two halves, and took the mean of the 1,000 calculated correlations as a measure of reliability of the mean image beauty rating across subsets of the population. Reliability was excellent, *R* = 0.976 (0.971 ≤ *R* ≤ 0.980 across the 1,000 split halves). These values are in the same range as those for the valence ratings for OASIS images and well above those for arousal ratings.

Second, to further probe agreement between participants, we calculated Spearman correlations between each participant’s and all other participants’ ratings (see also Wallisch and Whritner, 2017). The average correlation coefficient was moderate (*M* = 0.40, *Md* = 0.42), with large variation (*SD* = 0.16) and distributed as a shifted normal distribution with a long tail into the negative correlations. When we averaged all correlation coefficients for each participant, their distribution was no different (*M* = 0.41, *Md* = 0.43, *SD* = 0.10). Thus, most people (80%) have moderate correlation (0.2 < *r* < 0.5) with the population. There are only a few individuals (15%) whose average correlations exceed 0.5 and only a thin tail (5%) below 0.2 extending to negative correlations.

Third, to assess what percentage of variance in beauty ratings can be attributed to a shared taste component, we ran an additional short study on August 6, 2018, to collect repeated measures on two random subsets of 45 OASIS images each, 90 images in total (see Supplementary Table S1 for the list of images). Recruitment and procedures for this study were the same as for the main study. Each of the 59 participants (22 women, 37 men, *M*_age_ = 34.1, age range = 20–62) of this additional study rated one of the 45-image sets twice in two blocks. The order of images was randomized within each block.

The mean beauty ratings per image in the repeated measures study were highly similar to mean ratings in the main study, *r* = 0.89, RMSE = 0.37. The mean test–retest *SD* per participant ranged from 0.06 to 1.12, with an average of 0.31. Thus, the estimated *SD* per rating (*SD* divided by the square root of two) ranged from 0.04 to 0.79, with an average of 0.22. We consider the ratings given in the two separate studies comparable and therefore used intra-participant correlations of repeated measures to assess the proportion of variance of beauty ratings that can be attributed to shared taste, as Vessel et al. (2018) did.

The mean squared within-subject correlation was 0.77 in our repeated measures study. Thus 77% of the variance is repeatable and the remaining 23% is unrepeatable variance of rating. We next assess how much of the 77% repeatable variance is due to shared vs. individual taste. The mean squared across-subject correlations for the main vs. repeated-measures studies were similar: 0.19 vs. 0.23. Using the value for the main study, we infer that 19% of the variance can be attributed to shared taste, and the remaining 0.77–0.19 = 58% is repeatable but not due to shared taste. This pattern holds true if we look at data from the same population, i.e., the repeated measures study, alone, where 54% of the variance is repeatable but not due to shared taste, and 23% of the variance is due to shared taste. The percentage of repeatable variance that is due to shared taste also did not differ markedly between image categories (objects: 24%; animals: 17%; persons: 21%; scenes: 33%) and neither did the percentage of repeatable variance not attributable to shared taste (objects: 58%; animals: 72%; persons: 64%; scenes: 56%).

These percentages all reflect the proportions of explained variance relative to the total variance of the ratings. We also repeated the analyses used by Vessel et al. (2018) to infer the percentage of variance attributable to shared taste relative to the variance of ratings that is repeatable alone. To do so, we computed the inter- and intra-subject correlations. Their average squared values serve as estimates of overall variance explained by shared taste (inter-subject correlations), and by idiosyncratic taste (intra-subject correlations). The ratio between inter- and intra-subject correlations and the variance left unexplained gives an estimate of the relative contributions of shared and idiosyncratic taste, respectively.

This yielded values very close to the ones reported above, with (average squared inter-subject correlation/average square intra-subject correlation) = 0.19/0.77 = 25% shared taste using the original data from 757 participants without repeated measures and 0.23/0.77 = 30% using just the repeated measures data.

In sum, we find that 19% of the variance in OASIS beauty ratings is due to shared taste (mean rating of each image), 58% is due to idiosyncratic taste (repeatable ratings differing across observers), and the remaining 23% is due to variable rating (not repeatable). Thus, in beauty ratings of OASIS images, universal shared taste contributed only one third as much variance as personal idiosyncratic taste. Variance accounted for is *r*^2^, and correlation is *r*. Thus, these results indicate that beauty is 1.7 times more correlated with individual (*r* = 0.76) than with shared taste (*r* = 0.44).

## Discussion

This study has four goals: (1) to provide a first set of ratings of beauty for a large image set to complement the existing emotional measures of the same images, (2) to assess how much of the variance in beauty ratings is due to shared vs. idiosyncratic taste, (3) to describe the relation of beauty ratings to pleasure and arousal, and (4) to further test the link between beauty and pleasure by describing the relation between beauty and several indicators of low pleasure: anhedonia, low mood, and depression.

We find that 19% of the variance in OASIS beauty ratings is due to shared taste (mean rating of each image), 59% is due to idiosyncratic taste (repeatable ratings differing across observers), and the remaining 22% is due to variable rating (not repeatable). Thus, in beauty ratings of OASIS images, personal idiosyncratic taste contributed three times as much variance as universal shared taste. In other words, beauty is 1.7 times more correlated with individual (*r* = 0.76) than shared taste (*r* = 0.44). Results consistently indicate that the pleasantness of images is strongly positively linked (*r* = 0.75) with the beauty experienced from them and that only images with high average pleasure obtain high average beauty ratings more than rarely. Normally beautiful images elicit less intense beauty in people who are more anhedonic or in a worse mood, and more anhedonic people never give high beauty ratings. Yet, beauty ratings were unrelated to depression and there was no consistent relationship between mean beauty and arousal ratings.

### Beauty Ratings for OASIS

We here provide the first ratings on the key aesthetic dimension – beauty – for a large image set – OASIS (Kurdi et al., 2017). The 900 OASIS images provide a broad range of beauty intensities and participants were able to differentiate the intensity of felt beauty from diverse images well. The distribution of means and *SD*s followed the expected pattern. In split-halves testing across our population of observers, reliability for beauty ratings was excellent, about as high as those obtained by Kurdi et al. (2017) for valence of the same images. We did not find indications for meaningful differences in ratings according to major demographic variables. This tells us that mean ratings are consistent across subsets of the population. The ratings and findings reported here are not representative of the entire US American population but they are strictly comparable to Kurdi et al. (2017) population, which was recruited in the same manner, i.e., via Amazon Mechanical Turk. Although our mTurk sample, like Kurdi et al. (2017), was relatively liberal, white, highly educated, and high-income compared to the national average, we still obtained a more diverse sample closer to the overall United States population than we could have in a comparable lab study.

The only demographic effect was that, on average, women gave higher beauty ratings than men did to most images in the “nude couple” theme (12 of 14 images with the “nude couple” theme). If it holds up, this might be an interesting phenomenon for future study.

Each image’s average beauty rating accounts for 36% of variance of the individual ratings. And a linear combination of the average beauty rating by each observer and of each image accounts for half the variance. Thus, half the variance is accounted for by linear dependence on each image’s average across observers and each observer’s average across images. One might call these average taste and individual enthusiasm. In the timeless discussions about whether beauty is relative or absolute, it is sometimes claimed that statements about beauty are absolute and meant to apply to all people (especially for faces), while at other times individual differences are acknowledged, as in, “beauty is in the eye of the beholder”. Our results show contributions from both absolute and relative. Having obtained repeated measures for a representative subset of the OASIS images, we find that 58% of the variance in beauty ratings is due to individual taste while 19% is due to shared taste.

When people say that beauty is highly subjective, they typically are referring to art. However, we did not show art. We showed photos of everyday scenes, animals, objects, and people. Inter-individual differences in preferences are smaller for such natural stimuli than for more abstract ones (Vessel and Rubin, 2010; Leder et al., 2016; Vessel et al., 2018). The 25% of shared variance in beauty ratings according to the same analysis as Vessel et al. (2018) is well below the roughly 50% of shared taste reported for face images alone (Hönekopp, 2006) and in-between the 33% and the 8% Vessel et al. (2018) report for landscapes and art, respectively. Thus, the OASIS images fall in between the extreme categories of natural stimuli vs. art when it comes to the proportion of shared beauty taste. The moderately strong correlation between different participants’ ratings in our study (*Md* = 0.43) was also higher than previously reported for movies (*Md* = 0.27, Wallisch and Whritner, 2017).

Finding consistent mean ratings for the OASIS images makes the reported means useful for stimulus selection in future experiments. This does not mean that we should disregard beauty judgments for stimuli for which agreement is low, such as art. On the contrary, the divide in agreement between art and images like OASIS may help to distinguish different kinds of beauty, such as natural vs. artistic beauty, as has been suggested by philosophers (Rebec, 1905; Levinson, 2011).

### Beauty Correlates With Pleasure

Average beauty intensity was highly correlated with average pleasure in all picture categories (see Figures 3A,B), confirming the suggested tight link between beauty and pleasure (Kant, 1790/2000; Fechner, 1876; Santayana, 1896; Chatterjee, 2011; Leder, 2013) and previous empirical findings (Brielmann and Pelli, 2017; Brielmann et al., 2017; Vartanian et al., 2013). Yet, beauty cannot be equated with pleasantness. In our study, more than 40% of variance in beauty ratings remained unexplained by pleasure. It thus remains important to assess the experience of beauty separately from pleasure.

Unlike pleasure, arousal was only weakly associated with beauty, if at all (see Figure 3B). Given the known dissociation between pleasure and arousal (Posner et al., 2005), finding one in the association to beauty seems plausible. However, our OASIS study took only self-reports of arousal, and at a single time point. It remains open whether measuring development of arousal over time, or physiologically, might reveal a stronger relation to the beauty responses.

### Anhedonia and Mood, Not Depression, Reduce Intense Beauty

Our results show that depression *per se* does not limit people’s ability to appreciate beauty. This runs against the notion that depressed people see the world merely in negative terms, unable to notice positive things around them.

This finding may explain the seeming paradox that many successful artists suffering from major depressive disorders – including Ernest Hemingway, Virginia Woolf, Pablo Picasso, and Jackson Pollock – seem to have experienced beauty in their own work and that of others. Since major clinical depression is strongly associated with anhedonia, our finding that even our participants with the highest depression scores reported intense beauty experiences is indeed surprising. It is also encouraging for therapeutic application of aesthetic experiences to improve well-being (e.g., Lomas, 2016).

It is unlikely that we failed to find a relation between beauty and depression due to a lack of sensitivity of our depression measure, the PHQ-9. The PHQ-9 has repeatedly proven to be highly sensitive and reliable (Kroenke et al., 2001; Kroenke and Spitzer, 2002; Martin et al., 2006). It is one of the most widely used instruments for measuring depression in population surveys, as acknowledged by the Center for Disease Control and Prevention (Reeves et al., 2011). It is also unlikely that the absence of an association between depression and beauty ratings is due to a lack of severely depressed participants in our study, as we have data from seventy participants with signs of mild (*N* = 39) or severe depression (*N* = 31).

When we look more closely at symptoms of depression that define the common notion of depression, i.e., low mood and an inability to experience pleasure (anhedonia), however, we do find impairments in beauty experiences. The intensity of beauty experienced from normally beautiful images decreases with increasing signs for anhedonia and self-reported low mood. We found only weak to moderate correlations between scores for depression (PHQ-9) and our mood and anhedonia measures (TEPS). Thus, what has been measured here as depression needs to be considered separately from the popular notion of “depression,” which refers to a prolonged low mood devoid of pleasure. Anhedonia is not unique to depression; it occurs in people without clinical diagnoses (Harvey et al., 2007) and in other clinical populations, e.g., schizophrenia, unrelated to additional depressive symptoms (Pelizza and Ferrari, 2009). Our results are in line with other dissociations of depression and anhedonia, including the finding that altered brain activation in response to positive memories is associated with anhedonia, but not depression (Keedwell et al., 2005).

Theories for how the brain processes beauty may benefit from our finding of the relationship between anhedonia and the experience of intense beauty. Increased activity in the vmPFC has consistently been associated with the experience of beautiful stimuli (Kawabata and Zeki, 2004; Ishizu and Zeki, 2011; Vartanian et al., 2013; Pegors et al., 2015; Wang et al., 2015). The same region exhibits excess activity in patients with anhedonia (Keedwell et al., 2005). Our finding that anhedonia is associated with a decreased ability to experience beauty fits into this picture. It suggests that proper functioning of the vmPFC may be necessary for and not just a byproduct of the beauty experience.

Thus, we find that a generalized inability to experience pleasure transfers to the ability to experience beauty, i.e., an aesthetic pleasure. This complements the documentation of music-anhedonia, the isolated inability to experience pleasure from music (Mas-Herrero et al., 2014, 2018; Martínez-Molina et al., 2016, 2019; Mallik et al., 2017), which is associated with a more specific deficit in the connectivity between reward regions like the mPFC and auditory perceptual regions (Sachs et al., 2016). The hypothesis that intense beauty requires intense pleasure predicted a correlation between anhedonia and decreased ability to experience intense beauty. Whereas finding no correlation would have disproven it, actually finding the predicted correlation supports the hypothesis.

We find a similar decrease in reported beauty for normally beautiful images when we look at mood instead of anhedonia. The simplest explanation for the parallel findings would be that people who are anhedonic are generally in a low mood. Our data reject this explanation because the correlations between TEPS and mood scores were only weak to moderate. Another explanation for how mood affects aesthetic judgments is a “spillover” effect. Monahan et al. (2000) suggested that better mood can partially explain the mere exposure effect – neutral stimuli are liked more after many brief presentations. However, the spillover theory suggests that the beauty of every image should be enhanced or, if anything, that the greatest increase in beauty should have occurred for the on-average least beautiful images. We find the opposite, i.e., that the beauty of normally beautiful images profits the most from high mood. Thus, the spillover effect cannot explain our findings.

Leder et al. (2004) proposed a different explanation for how mood affects aesthetic ratings for art. They drew on the Forgas (1995) Affect Infusion Model, according to which a positive mood supports holistic processing. As holistic processing activates broader semantic networks, it might increase the intensity of experiences. On this account, participants in a better mood have a more intense beauty experience because they access richer associations when viewing beautiful images. As we used a unipolar scale that did not ask participants to think about the opposite of beauty, e.g., ugliness, intensity of the experience may have only risen for those images that have beauty-related associations. Framed in the negative, participants in a worse mood might be less able to experience beauty because a restricted mode of cognitive processing hindered them from experiencing intense beauty.

### Future Directions

We here present our findings as supportive of the notion that intense beauty requires intense pleasure. This empirical finding leaves open the question of whether the underlying mechanism distinguishes beauty from pleasure. An alternate interpretation is that beauty and pleasure are merely two judgments based on the same hedonic intensity and might differ solely in their thresholds, with a higher threshold for “beautiful” than for likable or pleasurable. Previous findings have suggested such a threshold mechanism (Brielmann and Pelli, 2017, 2018). It remains for future research on beauty to clarify whether or not beauty and pleasure experiences rely on the same hedonic intensity or separate albeit closely related ones.

By providing beauty ratings for a large (900 images) and openly accessible image database, we offer our colleagues in experimental aesthetics a valuable tool for stimulus selection, at least in terms of the most frequently named aesthetic adjective: beauty. Ratings on other dimensions of aesthetic experiences, such as interestingness (Berlyne, 1970; Cupchik and Gebotys, 1990; Silvia, 2010), may further enhance the selection of stimuli in empirical aesthetics. Moreover, relating ratings of the same images on different aesthetic dimensions may give us interesting insights into the composition of aesthetic experiences. Menninghaus et al. (2019a), for instance, compared beauty, sexiness, elegance, and grace in terms of how participants explicitly rated the *concepts*. The beauty ratings we provide for OASIS allows extension of such comparisons from the theoretical to the experiential level, i.e., to ask whether and for which stimuli different aesthetic ratings are the same or different.

Furthermore, we here assessed only the relation between beauty and a small, basic set of emotional experiences – pleasure and arousal. Future studies should broaden the emotional space investigated and look, for example, at other emotions (e.g., happiness, sadness, anger, fear, disgust, surprise) that can also be perceived in music (Mohn et al., 2011). Special attention has been given to the “sad music paradox” (e.g., Mohn et al., 2011; Vuoskoski et al., 2012; Lee et al., 2013; DeMarco et al., 2015). People like to listen to sad music, and they often do so when in a sad mood (Mohn et al., 2011; Vuoskoski et al., 2012). Perhaps beauty is fostered by congruency between stimulus- and mood-induced emotion. Another recently proposed idea is that the feeling of beauty is a distinct (aesthetic) emotion in and of itself (Menninghaus et al., 2019b). A database of beauty ratings for various images like ours provides a useful tool for testing whether the multiple components of aesthetic emotions are indeed part of feeling beauty as suggested by Menninghaus et al. (2019b).

We here assessed beauty as a unipolar construct, asking participants to rate its intensity. The unipolar assumption is supported by recent findings of a transcranial brain stimulation study (Nakamura and Kawabata, 2015). Suppression of left primary motor cortex and medial prefrontal cortex in this study affected beauty but not ugliness judgments, which dissociates ugly and beautiful as distinct dimensions. Yet, many other studies have employed a bipolar scale with the extremes beauty and ugliness (e.g., Kawabata and Zeki, 2004; Zeki et al., 2014; Muñoz and Martín-Loeches, 2015; van Paasschen et al., 2015). In future studies, it might be useful to also obtain ugliness ratings to shed more light on the emotional dimensionality of beauty and clarify whether it is a uni- or bipolar construct at the behavioral as well as neural level.

We found ratings to be highly consistent between groups of participants, indicating broad agreement on what is considered beautiful. Even though Amazon mechanical Turk provides a considerably broader spectrum of participants than studies in most laboratories, it would be interesting to probe agreement on beauty of the OASIS in the extremes: Specific subpopulations, such as artists, art historians, or art critics, may not agree with the average United States–American mTurk participant. We imagine that many of these people, whose profession is so tightly linked to beauty, might dismiss most of the highly rated OASIS images as kitsch or cliché. Cross-cultural comparisons of beauty ratings might further our understanding of universality vs. cultural specificity of beauty.

## Conclusion

We present: (1) reliable beauty ratings on a large and diverse image set that complement existing emotional ratings of the same images (the 900 OASIS images); (2) the finding that universal shared taste contributed only one third as much (19%) as personal idiosyncratic taste (58%) to the variance of beauty ratings; (3) a first general description of the relation between beauty and emotional responses, where beauty is highly correlated with pleasure (*r* = 0.75) but largely independent of arousal, and where only images with an average pleasure rating above 4 (of a possible 7) achieve beauty averages that often (>10%) exceed the overall median beauty (5.0); (4) further support for the notion that beauty is a kind of pleasure in that beautiful images lose their beauty with anhedonia (*r* ∼ 0.33) and that the most anhedonic participants’ average beauty ratings never exceeded the overall median.

## Data Availability Statement

All datasets generated for this study are included in the Supplementary Material and available at https://github.com/aenneb/OASIS-beauty.

## Ethics Statement

This study was carried out in accordance with the recommendations of NYU, UCAIHS (University Committee on Activities Involving Human Subjects; IRB-FY2016-404) with written informed consent from all subjects. All subjects gave written informed consent in accordance with the Declaration of Helsinki. The protocol was approved by the NYU, UCAIHS.

## Author Contributions

AB and DP conceptualized the experiments, and reviewed and edited the manuscript. AB acquired and curated the data, analyzed the data, and created the figures, tables, and the first draft of the manuscript. DP supervised the project.

## Conflict of Interest

The authors declare that the research was conducted in the absence of any commercial or financial relationships that could be construed as a potential conflict of interest.

## Footnotes

1 http://www.benedekkurdi.com/#oasis

2 https://github.com/aenneb/OASIS-beauty

## Supplementary Material

FIGURE S1 Matrix of raw data for all image sets. The data are sorted according to image category and name on the horizontal axis and according to the highest correlations between residuals of neighboring participants on the vertical axis. Lighter areas indicate higher ratings, darker areas lower ones. Margins indicate the mean rating per participant across all images along the vertical axis and mean beauty per image across participants along the horizontal axis.

FIGURE S2 Matrix of residuals for all image sets. Residuals are sorted according to image category and name on the horizontal axis and according to the highest correlations between residuals of neighboring participants on the vertical axis. Lighter areas indicate higher residuals, darker areas lower ones. Margins indicate the average absolute residuals across images per participant along the vertical axis and the *SD* of the residuals per image across participants along the horizontal axis.

TABLE S1 List of images used for repeated measures testing.

FILE S1 Instructions. Literal instructions presented to the participants.

FILE S2 Minor demographic effects. Results of the analyses regarding the effects of demographic variables other than gender.

FILE S3 Additional control analyses. Results of additional analyses of the first 100 trials per participant and separate analysis of the item 1.
