# Supplementary figures and images for: Intense Beauty Requires Intense Pleasure
Source: Front Psychol. 2019 Nov 5;10:2420. doi: 10.3389/fpsyg.2019.02420 (PMC6848232; doi:10.3389/fpsyg.2019.02420)

**Image set 1**

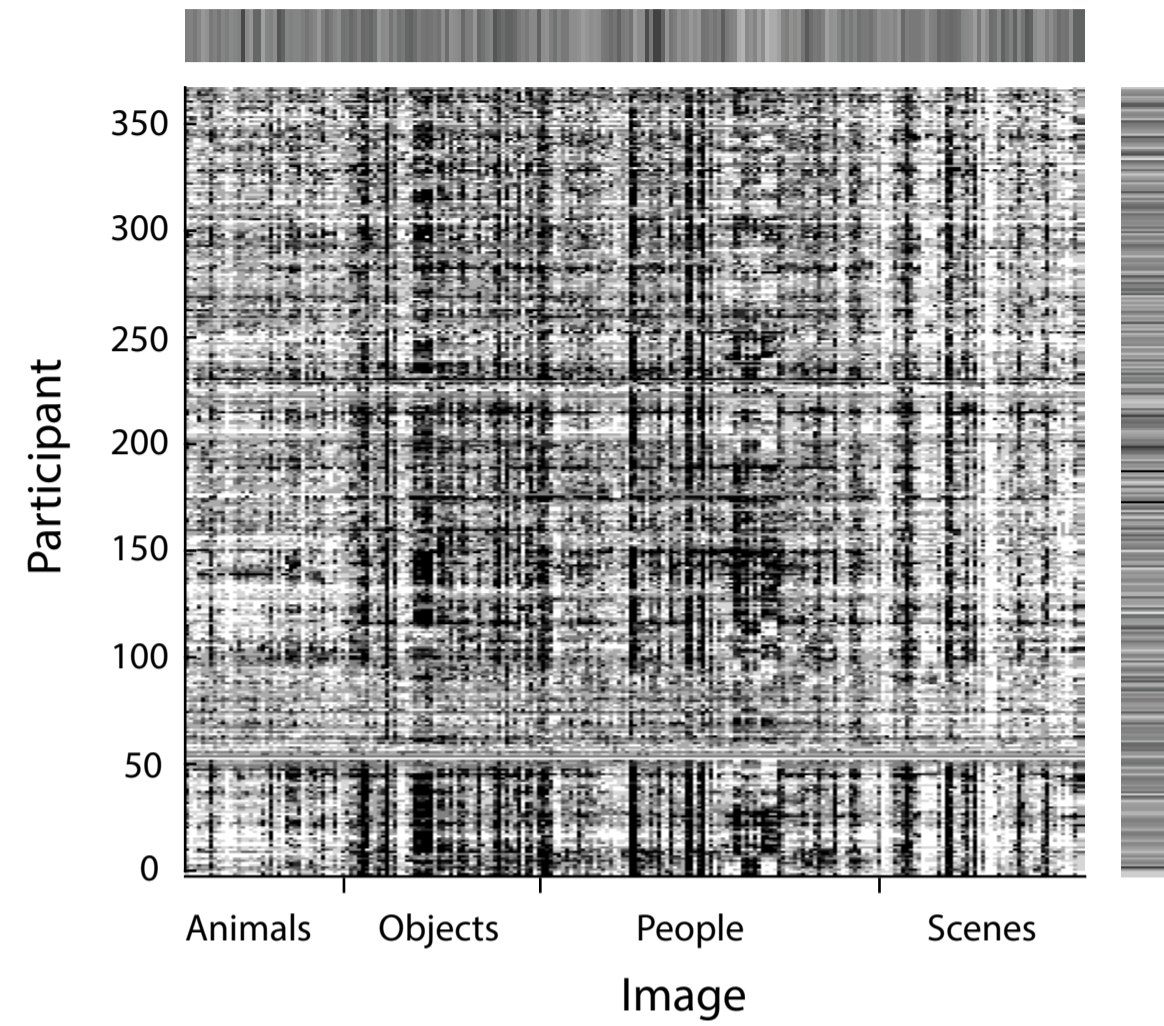

**Image set 2**

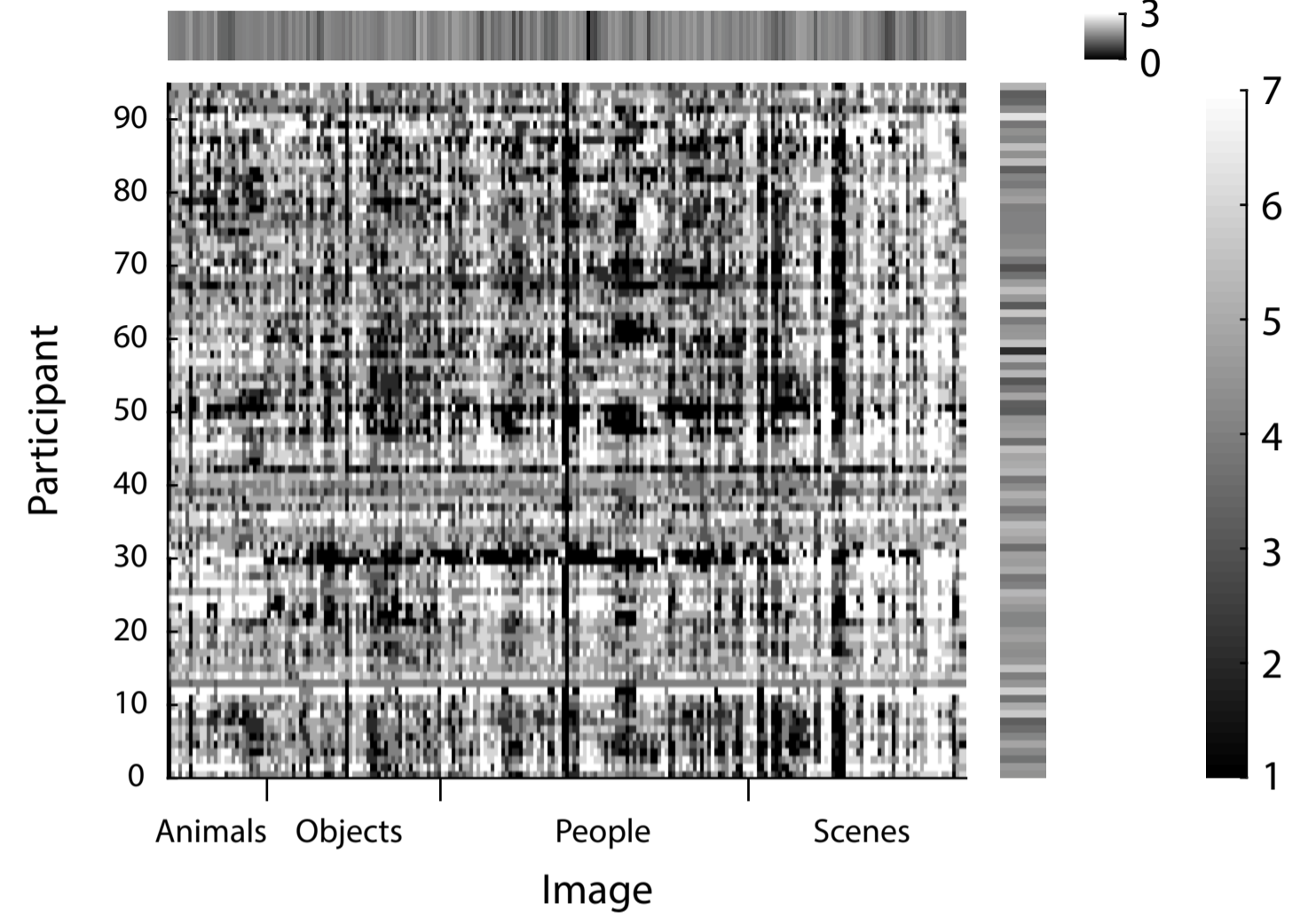

**Image set 3**

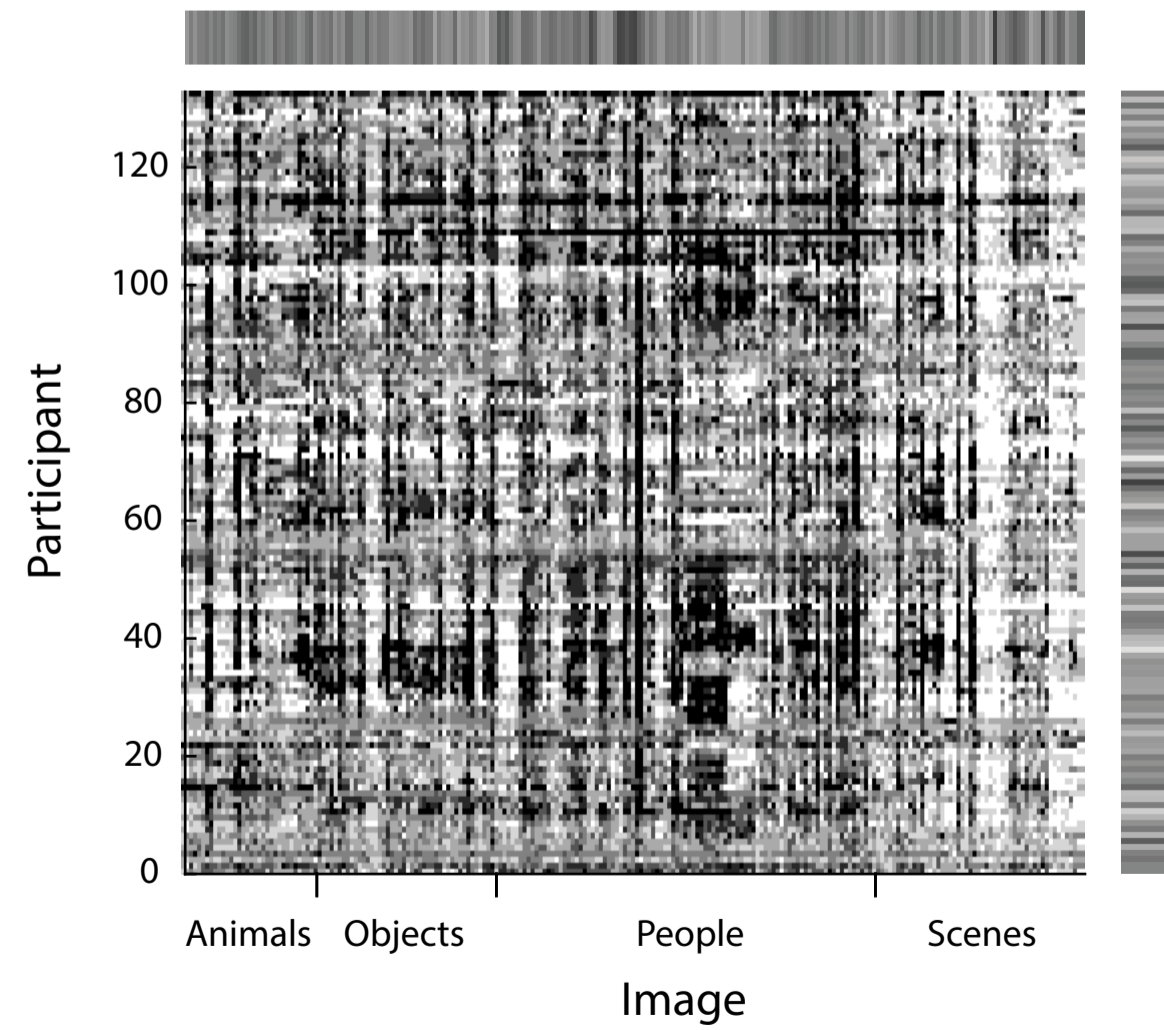

**Image set 4**

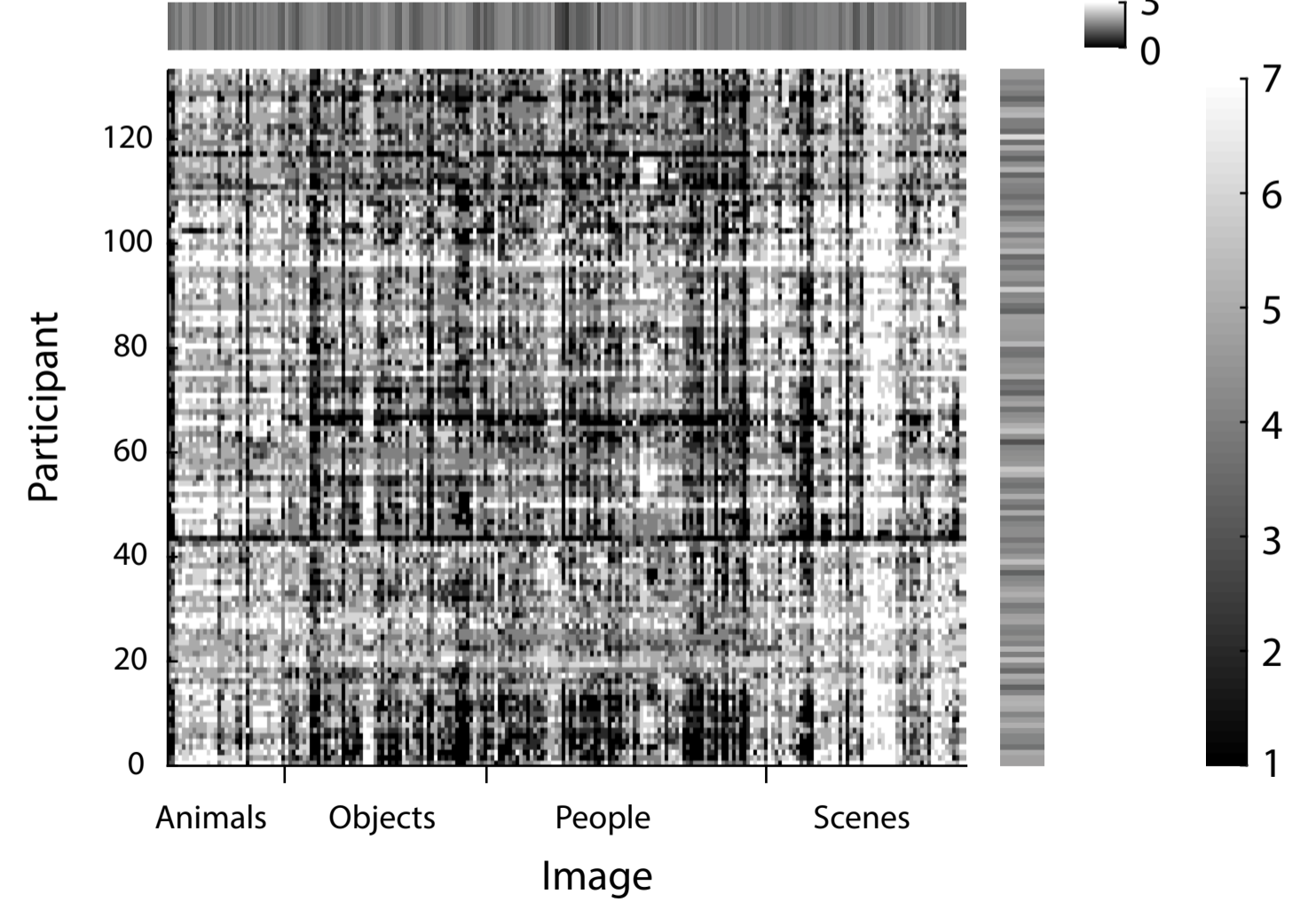

Supplement: FIGURE S1 — Matrix of raw data for all image sets. The data are sorted according to image category and name on the horizontal axis and according to the highest correlations between residuals of neighboring participants on the vertical axis. Lighter areas indicate higher ratings, darker areas lower ones. Margins indicate the mean rating per participant across all images along the vertical axis and mean beauty per image across participants along the horizontal axis. [file Image_1.pdf]

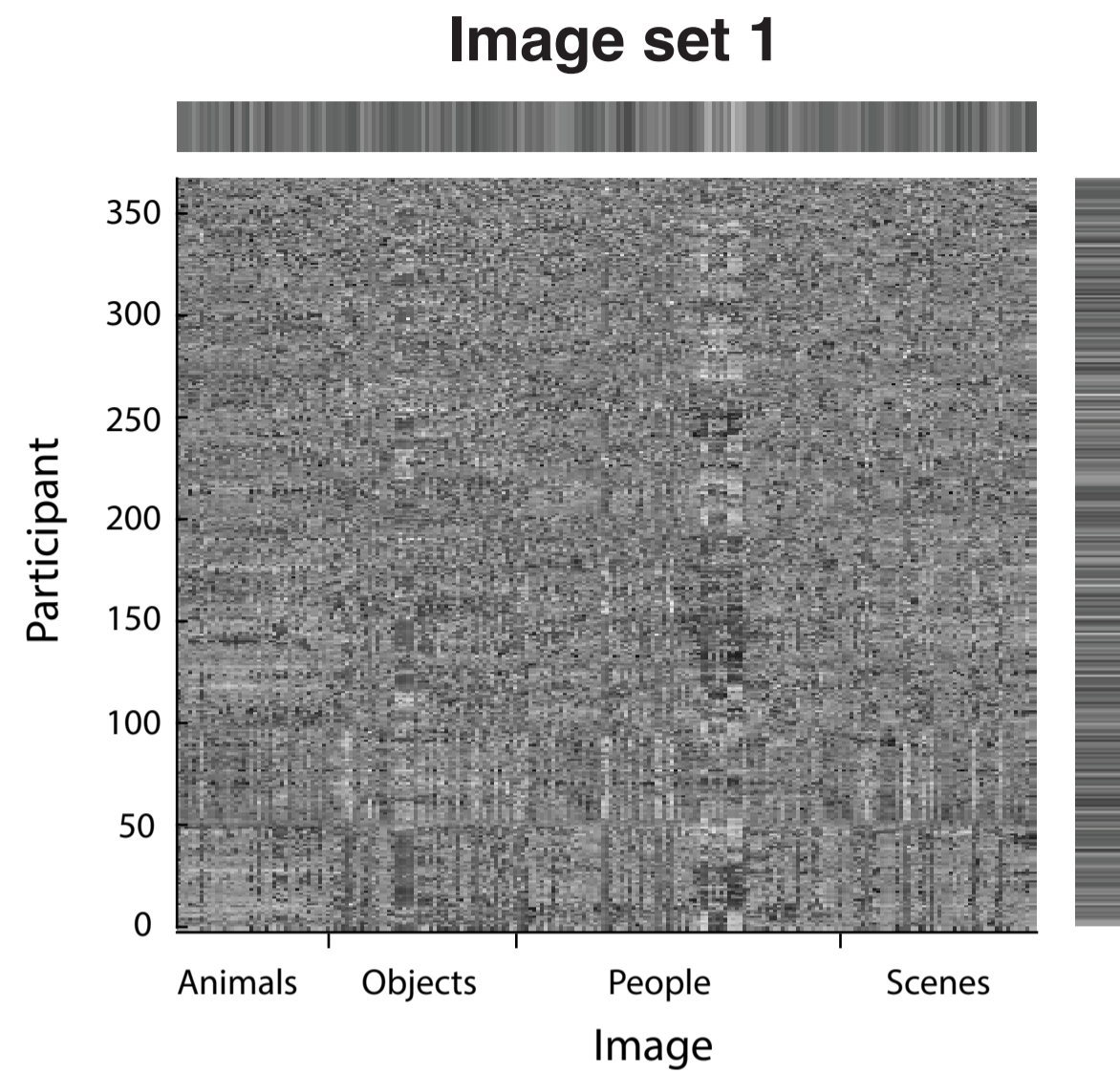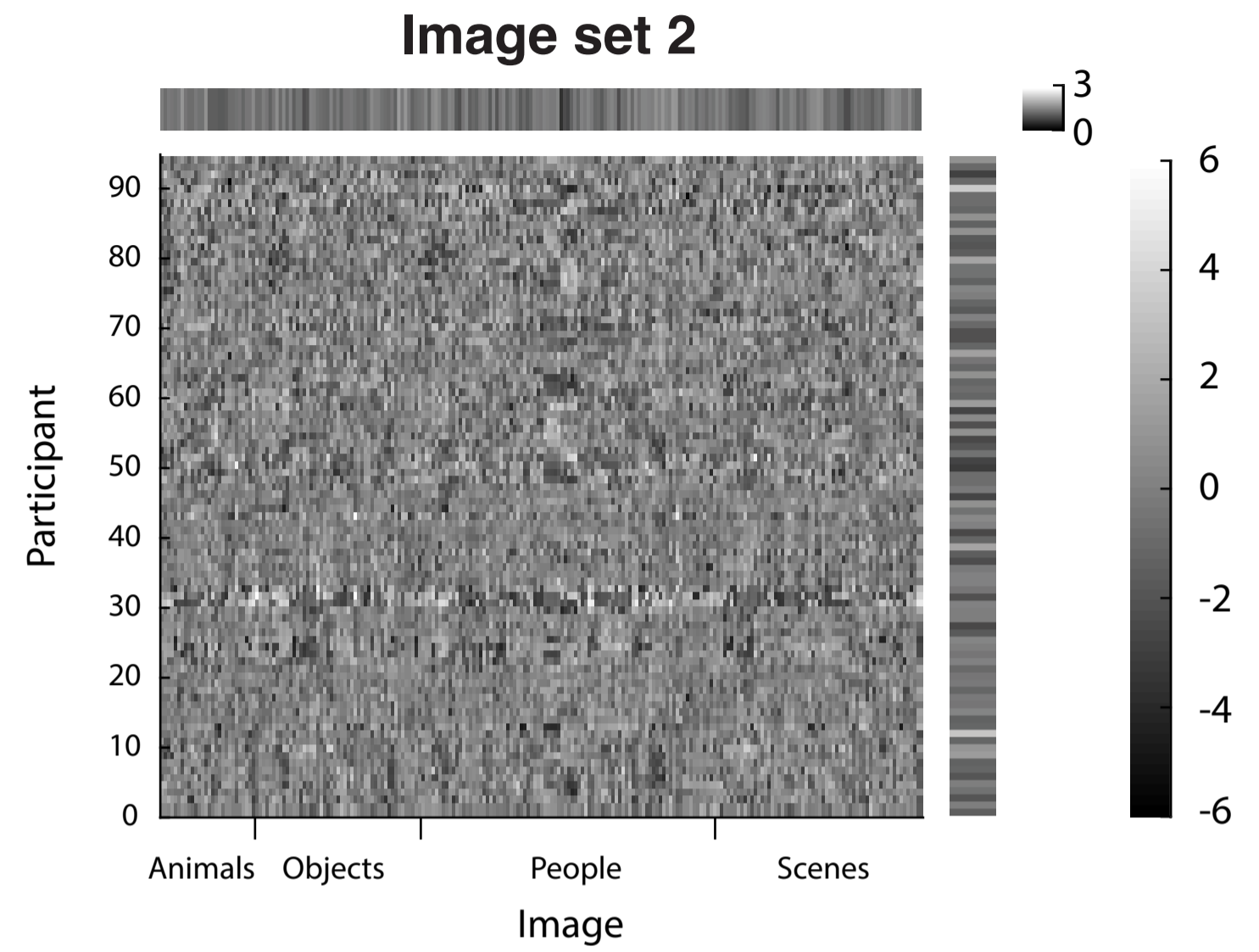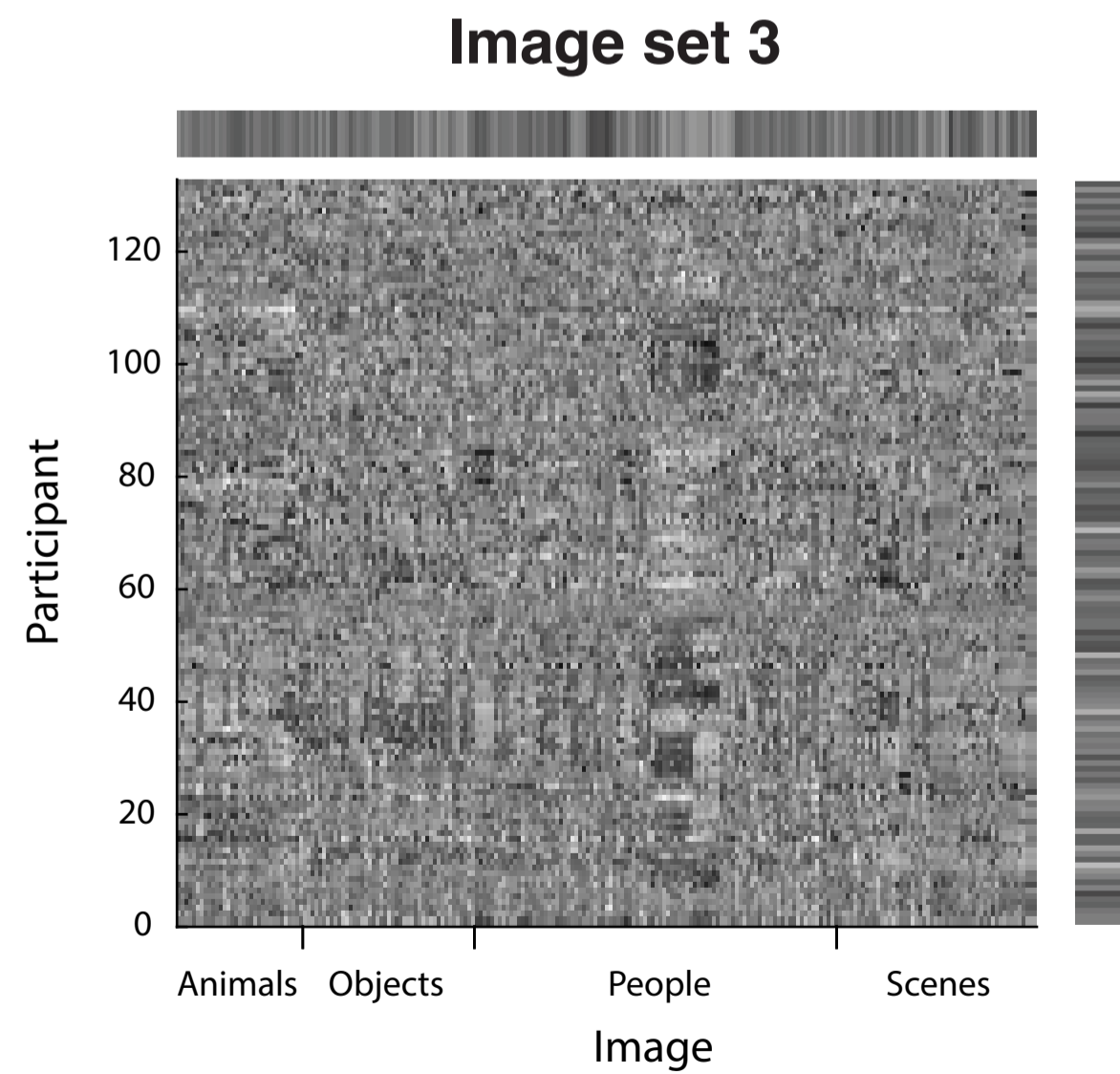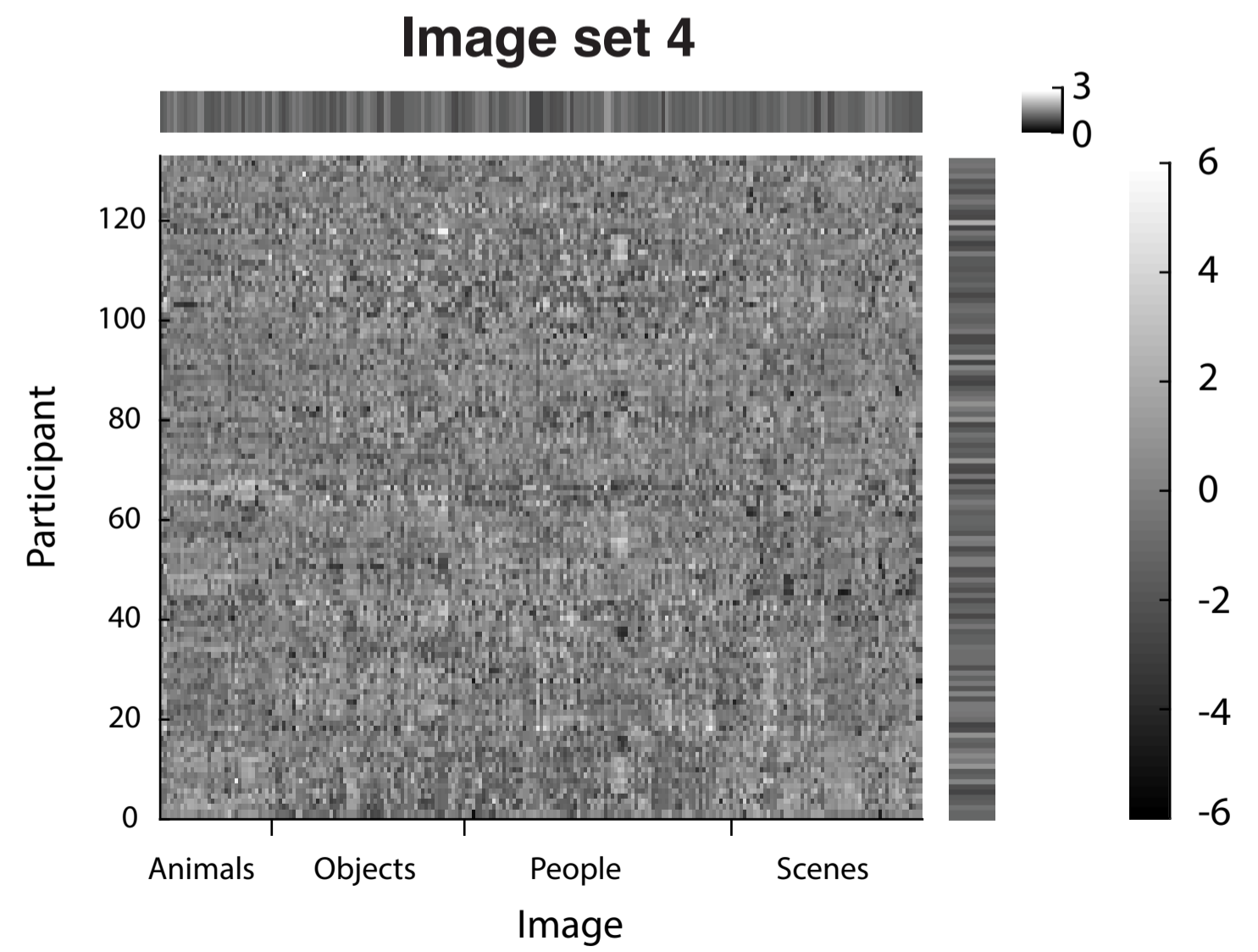

Supplement: FIGURE S2 — Matrix of residuals for all image sets. Residuals are sorted according to image category and name on the horizontal axis and according to the highest correlations between residuals of neighboring participants on the vertical axis. Lighter areas indicate higher residuals, darker areas lower ones. Margins indicate the average absolute residuals across images per participant along the vertical axis and the SD of the residuals per image across participants along the horizontal axis. [file Image_2.pdf]
