# Supplementary material for: Intense Beauty Requires Intense Pleasure
Source: Front Psychol. 2019 Nov 5;10:2420. doi: 10.3389/fpsyg.2019.02420 (PMC6848232; doi:10.3389/fpsyg.2019.02420)
Supplement: FILE S1 — Instructions. Literal instructions presented to the participants. [file Data_Sheet_1.PDF]

Intense beauty requires high pleasure

Aenne A. Brielmann & Denis G. Pelli

S1 file

Instructions

The following three instruction screens were presented to participants after informed consent:

Screen 1: "Pictures communicate. They depict people, objects, and scenes. In this study, we are interested in how various pictures make people feel. As you answer, please remember: There are no right or wrong answers. We are interested in your individual opinion. Please do not take too long over any picture. Your first response is as good as any."

Screen 2: "We will ask you to rate a series of pictures in terms of how much beauty they make you feel.
In other words, we would like to know how intense the feeling of beauty is that the picture evokes; whether it is good or bad doesn't matter.
Use the right side (bottom on a mobile device) of the scale to mark your answer if the picture makes you feel intense beauty.
Use the left side (top on a mobile device) of the scale to mark your answer if the picture makes you feel no beauty at all.
Use the middle of the scale to indicate that you are feeling moderate beauty, that is, halfway through the two extremes.
Please use the full range of the scale to make your responses rather than relying on only a few points."

Screen 3: "When making your ratings, you need to forget about whether the picture depicts something good or bad. We ask you to rate the picture only in terms of the intensity of beauty it makes you feel and not in terms of goodness or badness.

For instance, you might have two pictures—one depicting a landscape on a sunny summer day (something good) and another depicting a landscape during an autumn thunderstorm (something bad). You can give both images the same rating because they are both creating a similar level of beauty, even though the feelings are not the same. Your answers should be based only on how strong the feeling of beauty is the picture creates."
