# Supplementary material for: Intense Beauty Requires Intense Pleasure
Source: Front Psychol. 2019 Nov 5;10:2420. doi: 10.3389/fpsyg.2019.02420 (PMC6848232; doi:10.3389/fpsyg.2019.02420)
Supplement: FILE S2 — Minor demographic effects. Results of the analyses regarding the effects of demographic variables other than gender. [file Data_Sheet_2.PDF]

Intense beauty requires high pleasure

Aenne A. Brielmann & Denis G. Pelli

S2 file

Minor demographic effects

A complete analysis of differences in mean and *SD* between demographic groups reveals many small effects. Due to the large sample size, many of these small effects are statistically significant.

To assess differences between demographic groups in average beauty per image, we performed median splits of our data by age and income. Beauty ratings of younger and older participants were very highly correlated, $r(989) = 0.95$, $p < 0.001$, 95% CI [0.95, 0.96]. The difference in mean beauty per image between age groups was merely 0.06, $t(899) = -5.48$, $p < 0.001$, $SD = 0.36$. The variance in ratings per image was slightly higher for younger than for older observers, $t(899) = 19.40$, $p < 0.001$, mean difference (*MD)* = 0.11 ± 0.17.

Close correspondence was also seen in ratings of high and low income groups, $r(989) = 0.97$, $p < 0.001$, [0.97, 0.97]. Average beauty ratings across images were again hardly different between groups, $t(899) = -5.44$, $p < 0.001$, $MD = -0.05 \pm 0.28$, and so were *SD*s per image, t(899) = 8.34, $p < 0.001$, $MD = 0.05 \pm 0.17$. Political self-identification's potential impact was assessed by splitting the data into a "liberal" and a "conservative" group, omitting ratings from "neutral" participants. Again, correlation of average ratings was very high, $r(989) = 0.96$, $p < 0.001$, [0.95, 0.96]. Liberals gave on average slightly higher beauty ratings than

conservatives, $t(899) = 10.24$, $p < 0.001$, $MD = 0.11 \pm 0.34$, and *SD*s did not differ by political orientation, $p > 0.05$, $MD = -0.02 \pm 0.23$.

Unlike correlations for gender, no pronounced outliers were visible in the scatter plots for the other demographic groups.
