# Supplementary material for: Intense Beauty Requires Intense Pleasure
Source: Front Psychol. 2019 Nov 5;10:2420. doi: 10.3389/fpsyg.2019.02420 (PMC6848232; doi:10.3389/fpsyg.2019.02420)
Supplement: FILE S3 — Additional control analyses. Results of additional analyses of the first 100 trials per participant and separate analysis of the item 1. [file Data_Sheet_3.PDF]

Intense beauty requires high pleasure

Aenne A. Brielmann & Denis G. Pelli

S3 file

Analyses on first 100 trials

Distribution of beauty ratings

Equivalent to data from all trials, participants used the entire range of the 1 – 7 scale during the first 100 trials. Mean beauty ratings per image ranged from 1.0 to 6.81. The distribution of means was not normal according to Shapiro-Wilk test for normality but skewed to the left, $W = 0.98$, $p < 0.001$, parameter estimates for skewed normal distribution, $\omega = 1.74$, $\alpha = -2.40$ [40]. There is a quadratic relationship between *SD* and mean of the beauty rating, $R^2_{adj} = 0.59$, $p < 0.001$.

Beauty variance due to differences within and across observers

Reliability for the first 100 trials was highly similar to the values obtained across all ratings, $R = 0.951$ ($0.943 \leq R \leq 0.959$ across the 1,000 split halves).

We also again calculated Spearman correlations between each participant's and all other participants' ratings. The average correlation coefficient was moderate ($M = 0.43$, $Md = 0.45$), with large variation ($SD = 0.19$) and distributed as a shifted normal distribution with a long tail into the negative correlations. When we averaged all correlation coefficients for each participant, their distribution was no different ($M = 0.44$, $Md = 0.45$, $SD = 0.10$). Thus, most people (72%) have moderate correlation ($0.2 < r < 0.5$) with others. There are fewer individuals (26%) whose

average correlations exceed 0.5 and only a thin tail (3%) below 0.2 extending to negative correlations.

Beauty versus pleasure and arousal

Beauty was highly positively correlated with pleasure, $r(898) = 0.75$, $p < 0.001$, 95% CI [0.72, 0.78]. When using general models to explain mean beauty by pleasure, there was little difference between the linear and quadratic models, both $R^2_{adj} = 0.57$, despite a slight difference in corrected Akaike Information Criterion AICc = 10.74. Unlike that with pleasure, the positive correlation between beauty and arousal ratings was very weak, $r(898) = 0.15$, $p < 0.001$, [0.09, 0.22]. Introducing a quadratic term for predictions of beauty from arousal ratings slightly improved explained variance, quadratic $R^2_{adj} = 0.04$, vs. linear $R^2_{adj} = 0.02$, difference in AICc = 10.46.

Linear Model

We fit a linear model to predict individual participants' beauty ratings for each image from the observer and image averages,

$$B_{i,0} = -4.279 + 0.992\,\bar{B}_i + 0.984\,\bar{B}_o \quad (1)$$

where i = image index, and o = observer index. Each image's mean beauty $\bar{B}_i$ is a good predictor of its beauty rating, all by itself explaining 36% of the variance. Observer bias (mean beauty rating per participant, $\bar{B}_o$) alone explains 14% of the variance. Together, they account for 49% of the variance.

Nonlinear model with interactions

The regression in Eq. 2 below predicts beauty ratings based on the interaction of mean image beauty and anhedonia, mood, and depression, accounting for individual response biases.

$$B_{io} = -2.940 + 0.687\,\bar{B}_i + 0.960\bar{B}_o - 0.003\,mood_o - 0.173\,TEPS_{ant,o} - 0.049TEPS_{con,o} - 0.005PHQ_o + \bar{B}_i \times \left(0.001\;mood_o + 0.038\,TEPS_{ant,o} + 0.018\,TEPS_{con,o} + 0.001\,PHQ_o\right) \quad (2)$$

This model explains hardly any more variance than does Eq. 1, 50.96% vs 50.83%, $p < 0.001$. Unsurprisingly, beauty ratings increase with increasing image beauty. The model reveals important interactions: the increase of beauty ratings with mean beauty is amplified with heightened mood and TEPS scores (i.e. less anhedonic). Beauty ratings are overall unrelated to depression ($p = 0.183$).

Unpacking interactions within image bins of varying beauty

In line with the findings from the linear regression, depression was not associated with a change in beauty ratings, all correlation CIs included zero (**Fig 1B**). In contrast, we again find that beauty is consistently associated with the TEPS scores and mood (**Fig 1A,C-D**). The higher the beauty, the stronger the positive association with TEPS or mood.

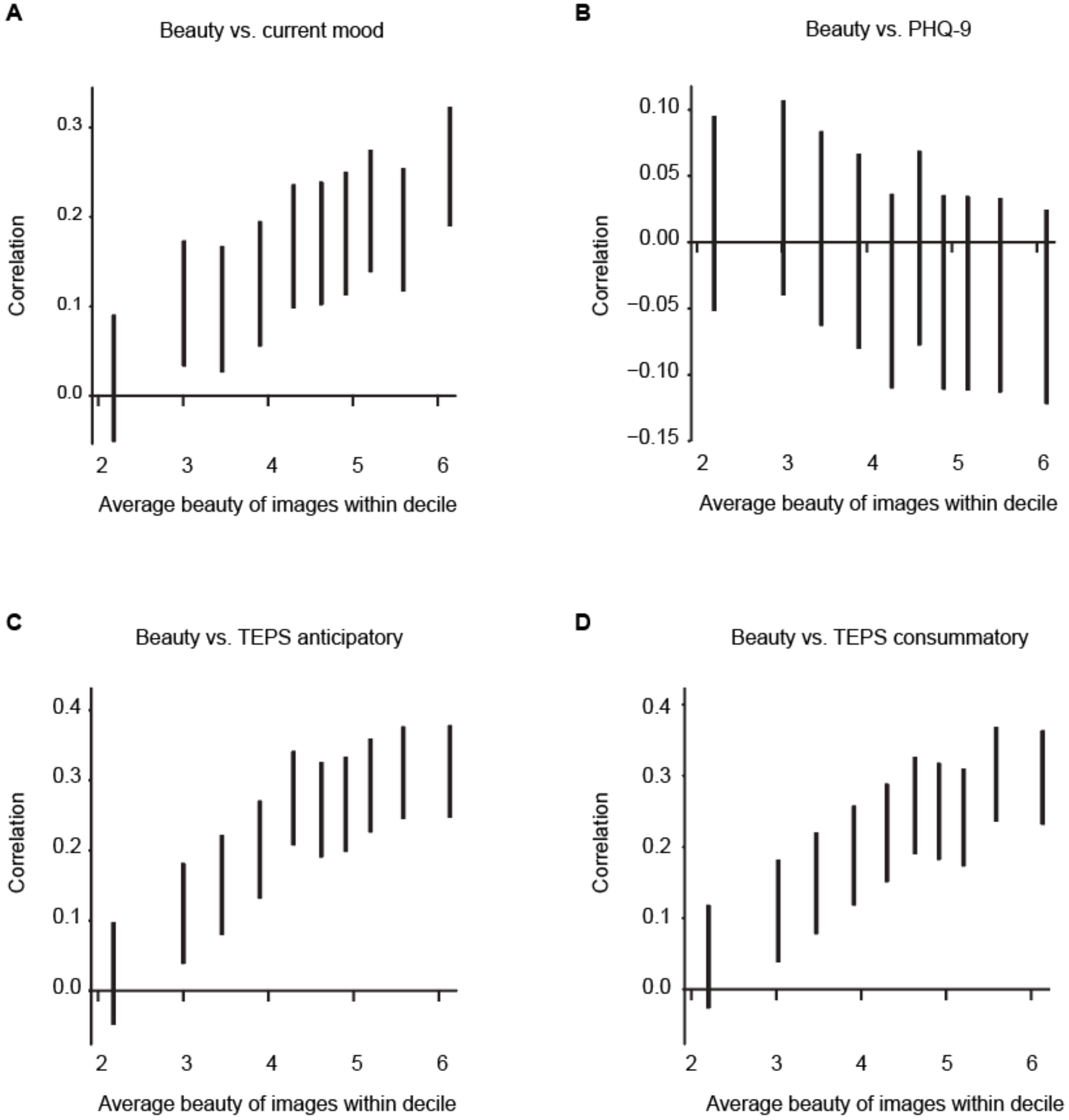

Fig 1. Vertical 95% confidence interval of the correlation coefficient of average beauty rating vs mood (A), PHQ-9 (B), TEPS anticipatory (C), and TEPS consummatory score (D) for data of the first 100 trials of each participant. The first decile represents the 10% of images that were rated lowest in beauty intensity, and so on. The horizontal scale is the average beauty of images in the decile.

Analyses of PHQ-9 question 1

The PHQ-9 contains one question "little interest or pleasure in doing things" that directly relates to one specific aspect of anhedonia. To assess whether we would obtain similar results as with the TEPS scores based on this question, we repeated the quantile-based analysis above based on that question alone. We find that this one question alone yields the same pattern of results as the analysis of the overall PHQ-9 scores (**Fig 2**).

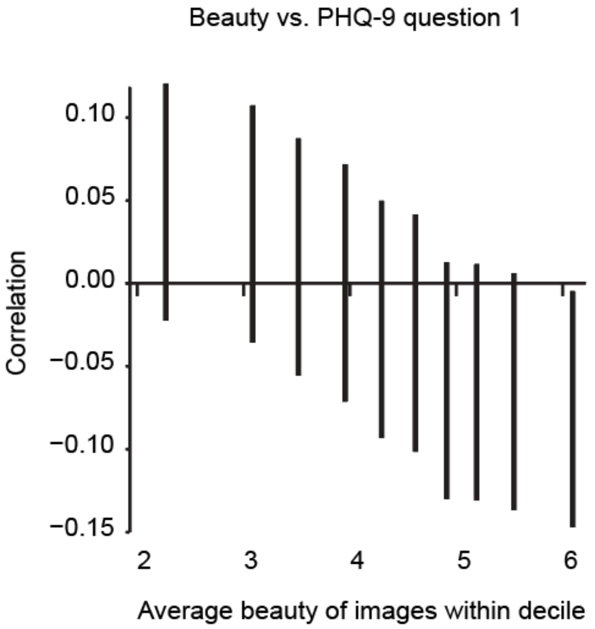

Fig 2. Vertical 95% confidence interval of the correlation coefficient of average beauty rating vs the first question of the PHQ-9 ("Little interest or pleasure in doing things"). The first decile represents the 10% of images that were rated lowest in beauty intensity, and so on. The horizontal scale is the average beauty of images in the decile.

Even though the PHQ-9 question analyzed here is related to anhedonia, its specificity differs from the TEPS. The PHQ-9 question is more specific about the time interval and less specific about the activity than TEPS. Firstly, the PHQ-9 asks about pleasure in the last two weeks, whereas the TEPS asks either about experienced or anticipated pleasure, i.e. past or future. Thus, the PHQ-9 may track a temporary mood, whereas the TEPS may assess a long-term trait. Secondly, the PHQ-9 question asks vaguely about pleasure "in doing things", whereas the

TEPS asks about pleasure for specific activities of daily life, e.g., tasting food. Both differences favor the interpretation of the results presented in the main manuscript whereby long-term trait anhedonia impairs the experience of beauty while current depressive symptoms do not.
